# *Empty Pericarp21* encodes a novel PPR-DYW protein that is required for mitochondrial RNA editing at multiple sites, complexes I and V biogenesis, and seed development in maize

**DOI:** 10.1371/journal.pgen.1008305

**Published:** 2019-08-02

**Authors:** Yong Wang, Xin-Yuan Liu, Yan-Zhuo Yang, Jin Huang, Feng Sun, Jishan Lin, Zhi-Qun Gu, Aqib Sayyed, Chunhui Xu, Bao-Cai Tan

**Affiliations:** 1 Key Laboratory of Plant Development and Environmental Adaptation Biology, Ministry of Education, School of Life Sciences, Shandong University, Qingdao, China; 2 Center for Genomics and Biotechnology, Haixia Institute of Science and Technology, Fujian Provincial Key Laboratory of Haixia Applied Plant Systems Biology, Fujian Agriculture and Forestry University, Fuzhou, China; University of Florida, UNITED STATES

## Abstract

C-to-U editing is an important event in post-transcriptional RNA processing, which converts a specific cytidine (C)-to-uridine (U) in transcripts of mitochondria and plastids. Typically, the pentatricopeptide repeat (PPR) protein, which specifies the target C residue by binding to its upstream sequence, is involved in the editing of one or a few sites. Here we report a novel PPR-DYW protein EMP21 that is associated with editing of 81 sites in maize. EMP21 is localized in mitochondria and loss of the EMP21 function severely inhibits the embryogenesis and endosperm development in maize. From a scan of 35 mitochondrial transcripts produced by the *Emp21* loss-of-function mutant, the C-to-U editing was found to be abolished at five sites (*nad7*-77, *atp1*-1292, *atp8*-437, *nad3*-275 and *rps4*-870), while reduced at 76 sites in 21 transcripts. In most cases, the failure to editing resulted in the translation of an incorrect residue. In consequence, the mutant became deficient with respect to the assembly and activity of mitochondrial complexes I and V. As six of the decreased editing sites in *emp21* overlap with the affected editing sites in *emp5-1*, and the editing efficiency at *rpl16*-458 showed a substantial reduction in the *emp21-1 emp5-4* double mutant compared with the *emp21-1* and *emp5-4* single mutants, we explored their interaction. A yeast two hybrid assay suggested that EMP21 does not interact with EMP5, but both EMP21 and EMP5 interact with ZmMORF8. Together, these results indicate that EMP21 is a novel PPR-DYW protein required for the editing of ~17% of mitochondrial target Cs, and the editing process may involve an interaction between EMP21 and ZmMORF8 (and probably other proteins).

## Author summary

Plant mitochondrial transcripts harbor hundreds of cytidine (C) that need to be converted to uridine (U) prior to translation. Typically, PPR proteins are recruited to specify the target Cs by binding to the upstream sequences of the edited site, and a given PPR is responsible for the editing of just one or a few sites, except atypical PPR-DYW protein DYW2 and MEF8, and P-class PPR NUWA. Here, we identified a canonical PPR-DYW protein EMP21 that is required for the editing of 81 mitochondrial target Cs. The editing is essential for mitochondrial complex assembly, and embryo and endosperm development in maize. In addition, the genetic assay showed that a portion (~30%) of the *rpl16*-458 sites are edited by EMP21 and EMP5 jointly. EMP21 and EMP5 directly interact with ZmMORF8. These findings revealed that the canonical PPR-DYW protein EMP21 plays a major role in the editing of a large number of sites in mitochondria, possibly by binding directly to the upstream sequences of certain edited sites or by recruiting other editing factors to the edited sites.

## Introduction

Plant mitochondrion possesses its own genome which retains ~5% genes from its prokaryotic ancestor. These genes encode proteins, ribosomal RNAs and transfer RNAs for oxidative phosphorylation, and protein translation. Plant mitochondria have acquired characteristic and complex RNA metabolism in the process of co-evolution with nucleus, including RNA cytidine (C)-to-uridine (U) editing, the splicing of introns, the maturation of transcript ends, RNA stabilization and RNA translation [1,2]. Numerous eukaryote-specific factors have been found to play vital roles in these processes. The pentatricopeptide repeat (PPR) proteins, which exceed 400 in many species, are one large family of these factors [3–6]. PPR proteins feature a tandem array of ~35-amino-acid repeat motifs [7], classified into P-class and PLS-class [4]. The P-class proteins harbor *bona fide* P-motifs with 35 amino acids, while the PLS-class ones comprise a mixture of P, L, and S motifs, where L motifs are 35–36 amino acids and S motifs are 31 amino acids [4,8]. Moreover, the PLS-class PPR proteins usually carry an E and/or DYW domain at their C-terminus. Many PPR proteins have been identified as being needed for the effective conversion of cytidine to uridine in both the chloroplast and the mitochondrion (reviewed in S3 Table), but the majority of them are responsible for editing at just one or a few sites.

RNA C-to-U editing is widespread in land plant organelles. Many angiosperms have more than 300 editing sites in mitochondria [9–12], and 20–40 editing sites in plastids [13–17]. RNA editing often restores evolutionarily conserved amino acids, generates start/stop codons, promotes intron splicing, and increases the efficiency of transfer RNA processing [18–24]. The process of cytidine to uridine conversion involves deamination [25,26], and the specific cytidine deaminase (CDA) has not been identified until recently [27]. This report found that two DYW domains containing the cytidine deaminase-like (CDAs-like) zinc binding signature residues (HxE(x)nCxxC) at the *Pp*PPR56 and *Pp*PPR65 C-terminus, respectively, function as cytidine deaminase in the C-to-U editing process. Most DYW domains in higher plant also harbor the CDAs-like signature residues, which are irreplaceable for RNA editing [28–30]. DYW1 is responsible for the editing of *ndhD*-1 site in Arabidopsis [31]. Mutation of the CDAs-like signature residues in DYW1 significantly decreases the zinc ion binding capacity, and abolishes the editing at *ndhD*-1 site [28]. Thus, DYW domains in higher plant probably have the same function as these domains in *Physcomitrella patens*.

In addition to the PPR proteins, C-to-U editing involves proteins from diverse families including multiple organelle RNA editing factors/RNA-editing factor interacting proteins (MORFs/RIPs), organelle RRM proteins (ORRMs), organelle zinc-finger 1 (OZ1), protoporphyrinogen oxidase 1 (PPO1), hydroxymethylbilane synthase (HEMC), tetratricopeptide repeat protein (WTG1) and chloroplast RNA helicase (ISE2). PLS-class PPRs dictate specificity by recognizing the approximately 5–20 nucleotides upstream of the editing sites [32–36]. MORFs/RIPs containing a conserved MORF/RIP motif are required for the editing of most sites in organelles. MORFs/RIPs have been reported to form hetero- or homo-dimer [37–39], and selectively interact with PPRs [40,41]. Four of ORRMs, ORRM2, ORRM3, ORRM4, and ORRM5, are required for the editing in mitochondria [42–44], and ORRM1 and ORRM6 function in the editing in plastids [45,46]. ORRMs can interact with RIPs/MORFs, and form hetero- or homo-dimer [42,43,46]. In Arabidopsis plastids, OZ1, a RanBP2-type zinc finger motif-containing protein, is responsible for editing at 30 sites, interacting with ORRM1 and RARE1, but not with any of the MORFs [47]. PPO1, a key enzyme for tetrapyrrole metabolism, was shown to play a surprising role in plastid RNA editing and interact with plastidial RIPs/MORFs, but not with PPRs [48]. *HEMC*, encoded a porphobilinogen deaminase, is associated with the RNA editing in plastids. AtECB2 (a PPR-DYW protein) directly interacts with HEMC, which in turn interacts with MORF8/RIP1 [49]. In chloroplast, the tetratricopeptide repeat protein WTG1 has been shown to involve the editing of at least two genes; it associates with both RIP1/MORF8 and MORF9 [50]. In addition, CP31, OCP3 and ISE2 influence the efficiency of cytidine to uridine conversion in plastids [51–54]. Recently, an active editing complex containing PPRs, RIP2, RIP9, RIP1, OZ1, ORRM1, and ISE2 was isolated from maize chloroplasts [55], lending convincing support for the hypothesis that the RNA C-to-U editing in plant organelles is carried out by an editosome.

Many PPR-DYW proteins have been reported to be required for the C-to-U editing in chloroplasts and mitochondria. But most of these proteins are responsible for the editing of just one or a few sites. Here we report a novel PPR-DYW protein EMP21 that functions in the editing of 81 sites in mitochondria. These editing events are crucial to the mitochondrial function and seed development in maize. Furthermore, we provide evidence that EMP21 may exert its function by interacting with ZmMORF8 (and probably other proteins).

## Results

### Phenotypic and genetic characterization of *emp21-1*

**Fig 1:**
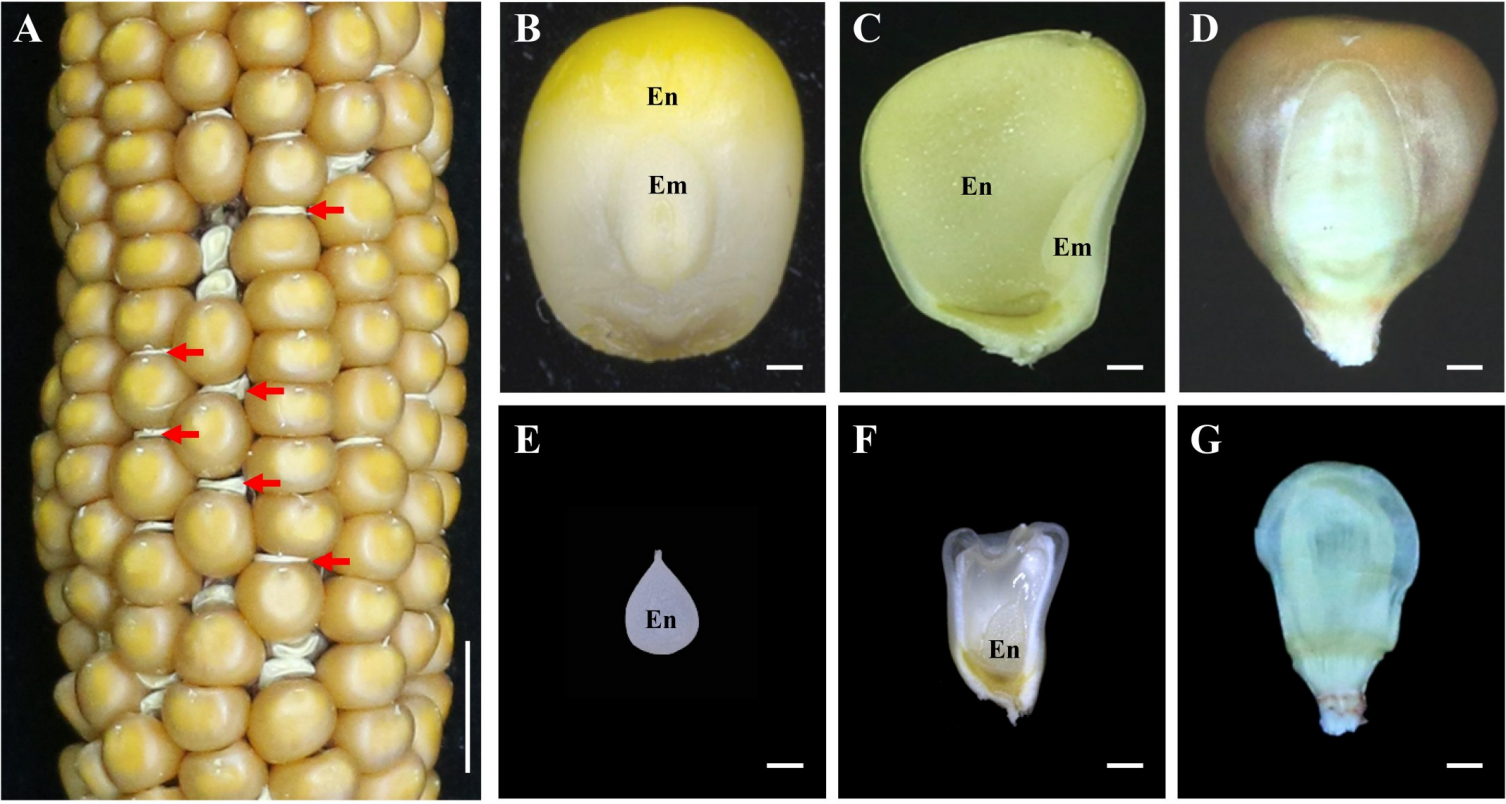
Embryogenesis and endosperm development are arrested in *emp21-1*. (A) A selfed ear of the *emp21-1* heterozygous plant. The empty pericarp kernels (*emp21-1*) are marked by red arrows. (B, C, E, and F) The embryo (em) and endosperm (en) of the wild type (WT) and *emp21-1* at 12 days after pollination (DAP). (D, G) The mature kernels of WT and *emp21-1*. (B-D), WT; (E-G), *emp21-1*. Bar = 1 cm in (A) and 1 mm in (B-G).

**Fig 2:**
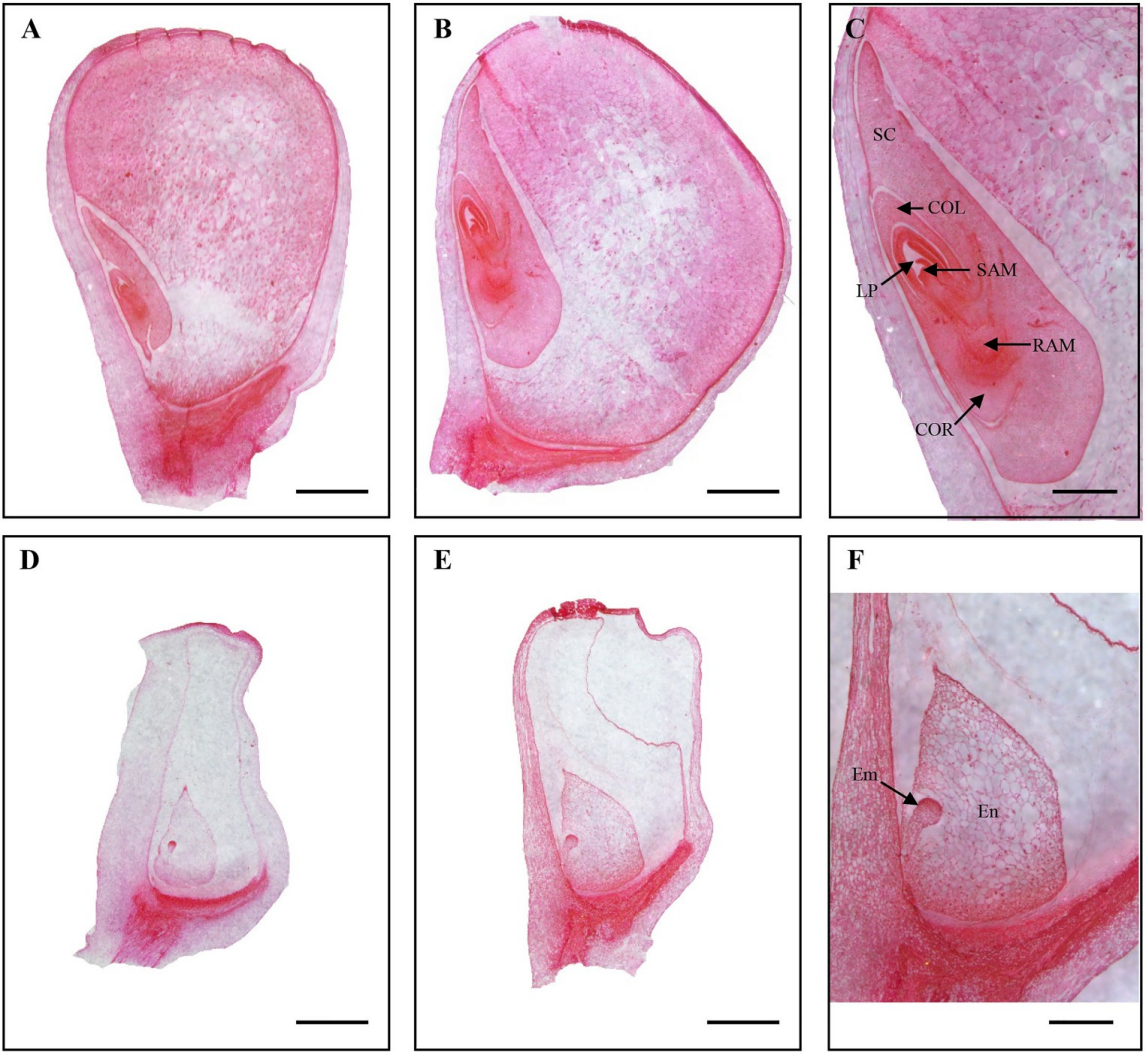
Mutation of *emp21-1* severely arrests maize embryogenesis and endosperm development. Paraffin section of (A-C) WT and (D-F) *emp21-1* developing kernels. (A, D) 12 DAP; (B, C, E, and F) 16 DAP. SC, scutellum; COL, coleoptile; LP, leaf primordia; SAM, shoot apical meristem; RAM, root apical meristem; COR, coleorhiza; Em, embryo; En, endosperm. Bar = 1 mm in (A, B, D, and E) and 500 μm in (C, F).

The *Mu* insertion mutant (*emp21-1*) was isolated from the UniformMu mutagenic population [56]. The selfed progenies of *emp21-1* heterozygotes segregated about 1/4 empty pericarp (emp) kernels (wild type: emp = 883:296 = 2.98:1, Fig 1A), indicating a nuclear and recessive mutation. The mutant kernels sampled at 12 days after pollination (DAP) were smaller than the wild type ones, and harbored a very much tiny embryo and a small transparent endosperm while the wild type kernels developed all structures (Fig 1B, 1C, 1E and 1F). At physiological maturity, the mutant kernels appeared shrivelled (Fig 1A and 1G). Hence, we named the mutant as *empty pericarp 21* (*emp21*). Inspection of sectioned tissue confirmed that both embryogenesis and endosperm development were defective in the mutants (Fig 2D–2F). While 12 DAP wild type embryos harbored a visible leaf primordium, a shoot apical meristem and a root apical meristem (Fig 2A), *emp21-1* embryos at this stage had only just reached the transition stage (Fig 2D). By 16 DAP, wild type embryos had entered the late embryogenesis stage (Fig 2B and 2C), but the *emp21-1* embryos remained at the transition stage and their endosperms were arrested at the cellularization stage (Fig 2E and 2F). Thus, loss of the *Emp21* function severely arrests embryogenesis and endosperm development in maize. The mutation proved to be embryo-lethal as all attempts to rescue them through *in vitro* culture failed.

**Fig 3:**
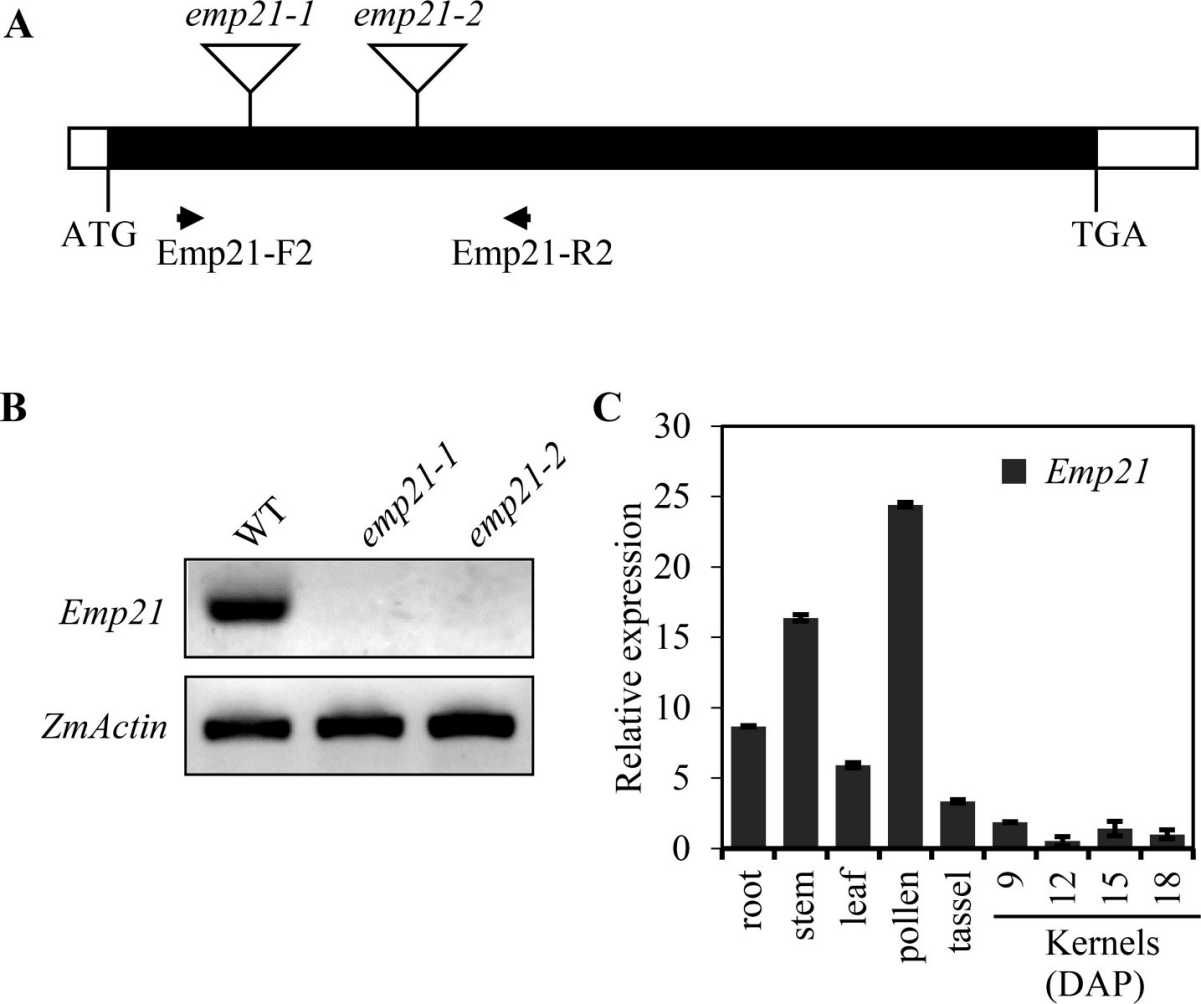
Transcription profiling of *Emp21* in wild type and the *emp21* mutants. (A) Gene structure of *Emp21*. The coding region is shown shaded, and the *Mu* insertions are marked by triangles. Primers (Emp21-F2/R2) were used for RT-PCR. (B) Transcription profiling of *Emp21* in the embryo and endosperm of 12 DAP kernels of WT and the two *emp21* mutants. The templates were normalized against *ZmActin*. (C) qRT-PCR analysis of *Emp21* transcription throughout the maize plant. Values shown are calculated from the mean of three biological replicates, and the error bars represent the ±SD.

A high throughput *Mu*-seq analysis [57] was used to identify the gene compromised in the *emp21-1* mutant. A *Mu* insertion at +280 bp from the translation start codon in GRMZM5G849971 was identified to be linked with the mutant (Figs 3A and S1A). A linkage analysis based on 46 segregants showed that the mutant phenotype is tightly linked with the *Mu* insertion (S1B Fig). To confirm that GRMZM5G849971 is the causal gene for the *emp21-1* phenotype, an independent insertion mutant was isolated from the UniformMu population. In this case, the *Mu* element is inserted at +608 bp from the GRMZM5G849971 translation start codon, designated *emp21-2* (Fig 3A). The selfed progenies of *emp21-2* heterozygotes segregated emp kernels similar to *emp21-1* (S2A Fig). Reciprocal crosses between *emp21-1* and *emp21-2* heterozygotes produced approximately 25% mutant kernels (S2B and S2C Fig). Thus, the *emp21* phenotype is caused by the mutation of GRMZM5G849971. Wild type *Emp21* transcripts could not be detected in either the *emp21-1* or *emp21-2* kernels (Fig 3B), suggesting that both alleles are likely null.

### *Emp21* encodes a mitochondrion-targeted PPR-DYW protein

**Fig 4:**
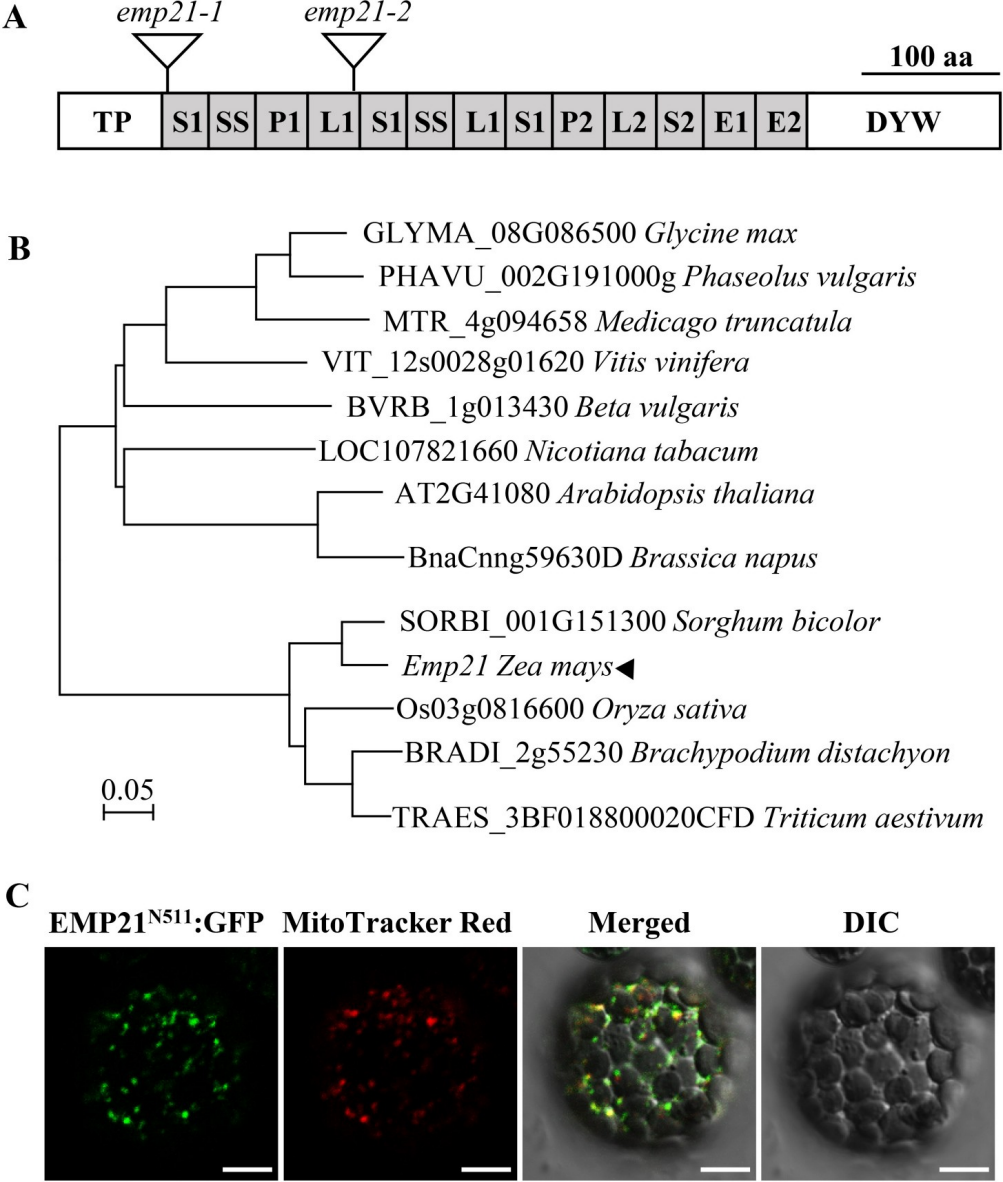
EMP21 is a mitochondrion-targeted PPR-DYW protein. (A) Protein structure of EMP21. EMP21 contains 11 PPR motifs, E1, E2, and DYW motifs. The *Mu* insertions are marked by triangles. (B) Non-rooted phylogenetic tree of EMP21 orthologs. (C) Subcellular localization of EMP21 in Arabidopsis protoplasts. The N-terminal 511 amino acid residues of EMP21 fused with green fluorescence protein (EMP21^N511^:GFP) was stably expressed in Arabidopsis. Florescence signals were observed by confocal microscope ZEISS LSM 880. The mitochondria were stained by MitoTracker Red. DIC, differential interference contrast. Bar = 10 μm.

*Emp21* encodes a canonical DYW-subclass PPR protein, consisting of 647 amino acid residues (Fig 4A). Based on the redefined PPR motifs [8], EMP21 possesses 11 PPR motifs, E1, E2 and DYW motifs (Figs 4A, S3 and S4). The DYW-motif in EMP21 contains the conserved CDAs-like signature residues (HxE(x)nCxxC) (S4 and S5 Figs). A phylogenetic analysis revealed extensive conservation in the sequence across both mono- and dicotyledonous species (Fig 4B). The EMP21 orthologs in *sorghum bicolor* (SbEMP21, SORBI_3001G151300), *Triticum aestivum* (TaEMP21, unnamed protein product) and *Oryza sativa* (OsEMP21, Os03g0816600) share 91.2%, 79.7% and 73.3% sequence identity with the maize EMP21, respectively (S4 Fig).

No target peptide was predicted for EMP21 according to the TargetP (http://www.cbs.dtu.dk/services/TargetP) and Predotar algorithms (https://urgi.versailles.inra.fr/predotar/predotar.html). To localize EMP21, the full-length EMP21 (without stop codon) was fused with the green fluoresent protein (GFP) and transformed Arabidopsis. Fifteen transgenic lines were generated, but none of them showed GFP signal. We suspected that EMP21-GFP may be too large to be efficiently expressed, or over-expression of the full-length protein may be detrimental to the cell. Then, the N-terminal region containing all the PPR motifs and E1, E2 motifs of EMP21 was fused with GFP and transformed Arabidopsis. Twenty transgenic lines were isolated and all showed GFP signals. The GFP signals were found in punctated dots that merged with the mitochondria which were stained by the MitoTracker Red (Fig 4C). No GFP signal was detected in chloroplasts or other compartments in the cell (Fig 4C), indicating that EMP21 is exclusively targeted to mitochondria.

Quantitative real-time PCR (qRT-PCR) assay indicated that *Emp21* is ubiquitously transcribed throughout the maize plant, with a relatively high level of expression observed in root, stem and pollen, and low expression in leaf, tassel and developing seeds (Fig 3C). Thus, *Emp21* is not a seed specific gene, but rather a constitutive gene that may have an essential role throughout plant growth and development. Because *emp21* is embryo-lethal, these impacts cannot be assessed.

### EMP21 is required for the C-to-U editing at 81 mitochondrial target Cs

The maize mitochondrial genome is predicted to harbor 35 protein-encoding genes including 22 genes of electron transport chain, 11 ribosomal protein genes, one maturase gene (*matR*), and one transporter gene (*mttB*) [58]. The Arabidopsis and rice mitochondrial transcripts harbor over 600 and 490 editing sites [9,12,59], whereas the maize editing sites in mitochondrial transcripts were only analyzed by direct sequencing of the RT-PCR amplified transcripts [60]. We used the strand- and transcript-specific RNA-seq (STS-PCRseq) method to analyze the editing sites in these 35 mitochondrial transcripts [59]. Based on a total of 600 Mb sequence data, 493 C-to-U editing sites were identified in these transcripts in maize (Table 1, S1 and S2 Dataset). Among those sites, 12 sites are edited 100%, 72 sites 99–100%, 170 sites 90–99%, 154 sites 50–89%, and 85 sites less than 50% (S3 Dataset). Most of these editing events cause alteration of the encoded amino acids (S3 Dataset).

**Table 1:** The target Cs in maize mitochondrial genes and the effect of the loss of *Emp21* function on their editing.

	*emp21*					WT
	Abolished	Decreased	Increased	Unaffected	Total	
complex I	2	26	6	155	189	189
*cob-*complex III	0	1	1	15	17	17
complex IV	0	12	1	25	38	38
complex V	2	2	0	43	47	47
cytochrome *c* biogenesis	0	21	5	82	108	108
ribosomal protein	1	12	9	54	76	76
*matR*	0	1	0	12	13	13
*mttB*	0	1	0	4	5	5
Total	5	76	22	390	493	493

**Fig 5:**
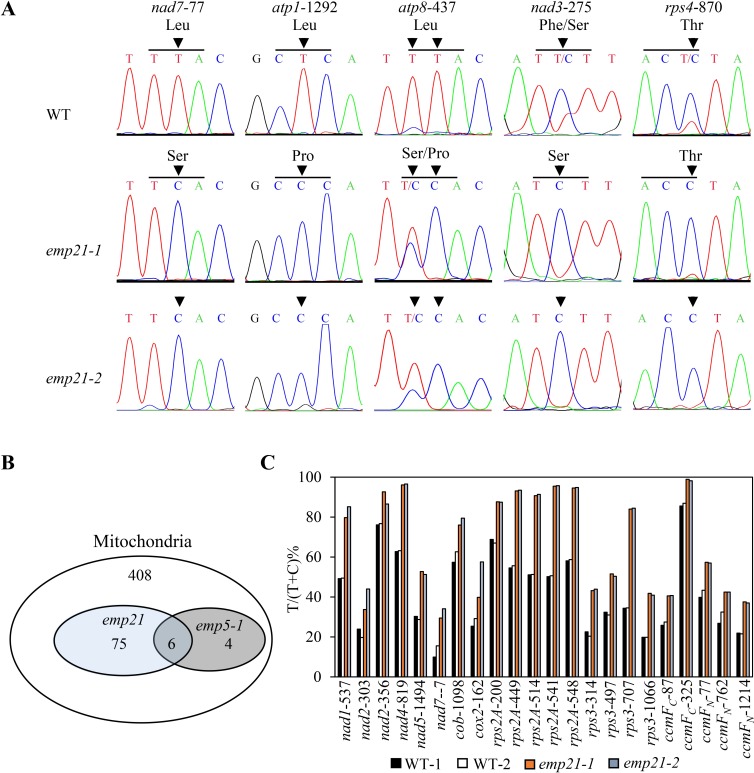
The editing at about 17% mitochondrial target Cs is defective in the *emp21* mutants. (A) The abolished editing sites in the *emp21* mutants. Sites subject to defective editing marked by arrows. The residues shown on the left are generated by an edited codon and those on the right by a non-edited codon. (B) The number of target Cs in WT maize mitochondria and of the abolished and decreased editing sites in the *emp21* and *emp5-1* mutants [18]. (C) Sites in the *emp21* mutants at which editing was more effective than in the WT. The relevant raw data are reported in S1 and S2 Dataset.

Since most of the known DYW-subclass PPRs function in the RNA C-to-U editing (reviewed in S3 Table), the STS-PCRseq method and direct sequencing of the RT-PCR amplified transcripts [18] were used to assess the editing profiles of the 35 mitochondrial protein-coding genes between *emp21* and wild type. These results revealed that the editing is completely abolished at the *nad7*-77, *atp1*-1292, *atp8*-437, *nad3*-275 and *rps4*-870 sites in both the *emp21-1* and *emp21-2* mutants (Fig 5A and S2 Dataset). The first three sites are fully edited in wild type, whereas the last two sites are edited at a 15% and 21% level in wild type, respectively. In addition, the editing at a further 76 sites, distributed in 21 transcripts (*nad2*, -*3*, -*4*, -*6*, -*9*, *cob*, *cox3*, *atp8*, *rps1*, -*3*, *-4*, -*7*, *-12*, -*12-ct*, -*13*, *rpl16*, *ccmB*, -*F*_*C*_, -*F*_*N*_, *matR* and *mttb*), was substantially reduced in the mutants (Table 1, S6 Fig and S2 Dataset), and the editing of 22 sites in 11 transcripts (*nad1*, *-2*, *-4*, *-5*, *-7*, *cob*, *cox2*, *rps2A*, *-3*, *ccmF*_*C*_, and *-F*_*N*_) (Table 1, Figs 5C and S7 and S2 Dataset) was substantially increased in the *emp21* mutants in comparison with the wild type. Increased editing has been reported in several mutants, such as *emp5*, *emp7* in maize, and *mef8*, *orrm5* in Arabidopsis [18,44,61,62]. However, decreased editing in such a large number of sites for a typical PPR-DYW gene mutant has not been reported previously.

Based on codes defined by the combinatorial residues at residue 6 of one PPR repeat and residue 1’ of the next PPR repeat [63–65], the EMP21 PPR motifs were largely aligned with the sequence upstream of *nad7*-77, *atp1*-1292 and *atp8*-437, but less aligned with those of *nad3*-275, *rps4*-870 and the other 76 sites where editing was compromised in the absence of EMP21 (S1 Table). It is possible that EMP21 recognizes *nad7*-77, *atp1*-1292 and *atp8*-437 by direct binding to the sequences, but through other means on the other 78 edited sites.

### The Nad7 Leu^26^ and Atp1 Leu^431^ residues are conserved across species

**Fig 6:**
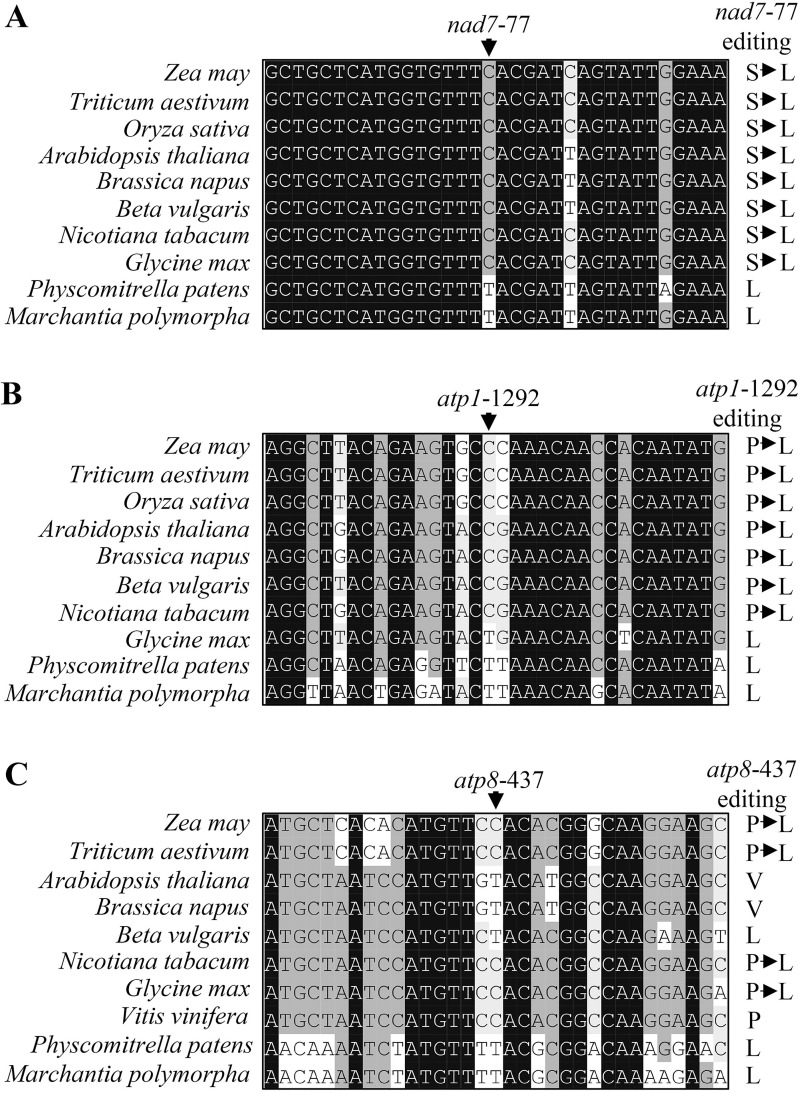
The Nad7 Leu^26^ (L^26^) and Atp1 Leu^431^ (L^431^) residues are highly conserved, while the residue at Atp8-146 are variable. (A-C) Alignment of the neighboring gDNA sequences of *nad7*, *atp1* and *atp*8. The nucleotide sequences and cDNA sequences were derived from GenBank/EST and GenBank/EMBL databases. The sites at which editing is abolished in the *emp21* mutant are arrowed.

Deficient editing at most of the sites requiring EMP21 resulted in a change in the encoded amino acid residues, for example Leu^26^ to Ser^26^ in Nad7, Leu^431^ to Pro^431^ in Atp1, and Leu^146^ to Ser^146^/Pro^146^ in Atp8 (Fig 5A). A comparison of both the gDNA and cDNA sequences of the orthologs of *nad7*, *atp1* and *atp8* suggested that Leu^26^ in Nad7 and Leu^431^ in Atp1 are conserved in both lower and higher plants (Fig 6A and 6B), while the amino acid residues at Atp8-146 encoded by *atp8*-437 have diverged markedly (Fig 6C). The amino acid residue at Atp8-146 is a Leu in *Zea mays*, *Triticum aestivum*, *Glycine max*, *Beta vulgaris*, *Nicotiana tabacum*, *Physcomitrella patens*, and *Marchantia polymorpha*, but a Pro in *Vitis vinifera* and Val in *Arabidopsis thaliana* and *Brassica napus* (Fig 6C).

### EMP21 is important for the assembly and function of mitochondrial complexes I and V

**Fig 7:**
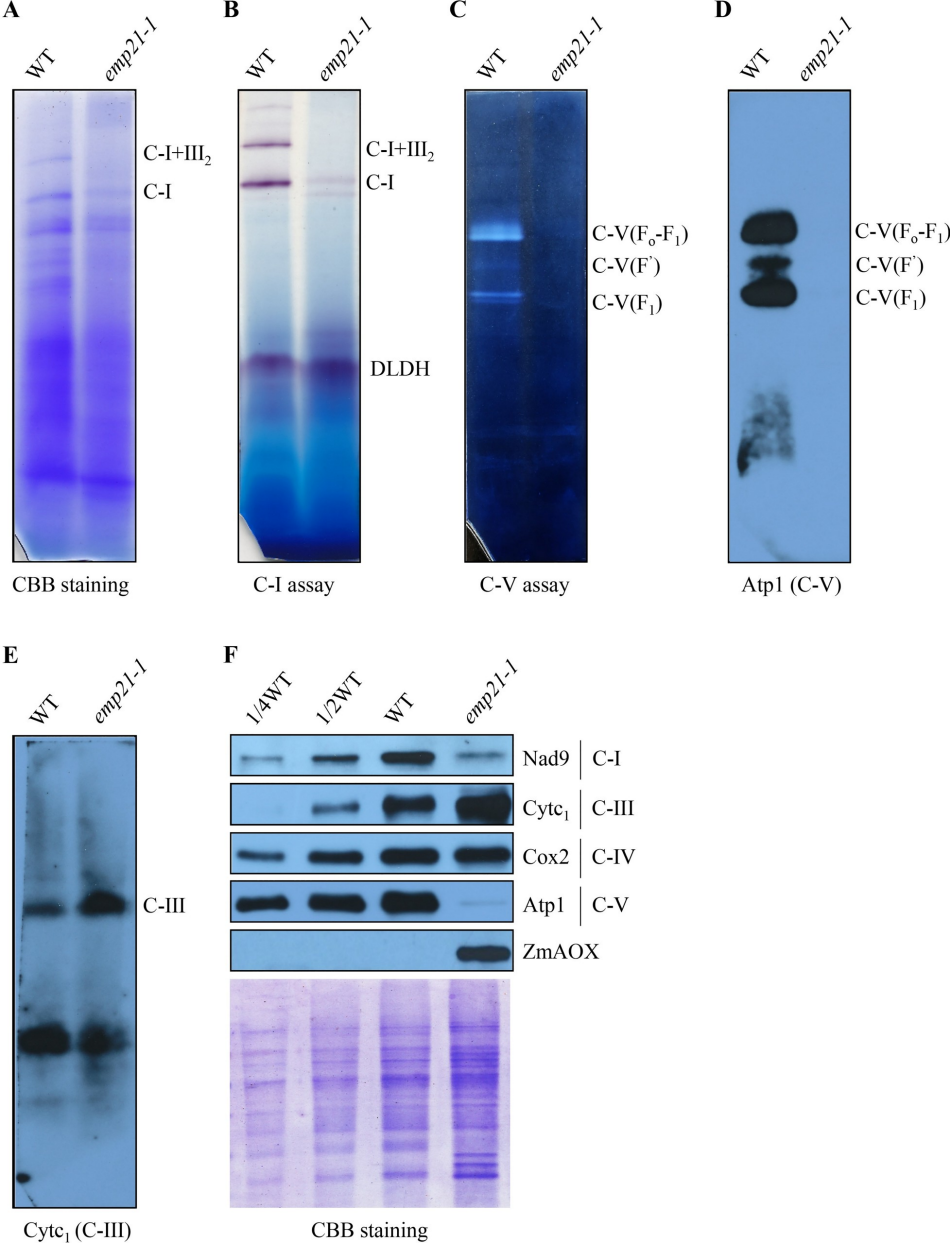
The assembly and activity of the mitochondrial complexes I and V are compromised in the *emp21-1* mutant. (A) Polyacrylamide gel electrophoresis (BN-PAGE) analysis of the assembly of complex I and supercomplex I +III_2_. The gel was stained with Coomassie Brilliant Blue (CBB). (B) In gel assay of the NADH dehydrogenase activity displayed by complex I. Dihydrolipoamide dehydrogenase (DLDH) activity was used as the loading control. (C) In-gel assay for F_1_F_o_-ATP hydrolyse activity of complex V. (D, E) Western blot analysis based on antibodies recognizing Atp1 (complex V) and Cytc_1_ (complex III). (F) Western blot analysis with antibody against Nad9, Cytc_1_, Cox2, Atp1 and ZmAOX. A gel stained with CBB was used for loading control. C-I: complex I, C-I+III_2_: supercomplex I +III_2_, C-V: complex V.

The defective editing in *emp21* occurs in the genes encoding the subunits of four mitochondrial respiratory chain complexes (complex I, III, IV, and V). The impact of the *Emp21* mutation on the assembly and function of the mitochondrial respiratory chain was investigated through the use of Blue Native-PAGE (BN-PAGE). The abundance of complex I in *emp21-1* was greatly reduced, while that of supercomplex I+III_2_ was below the level of detection (Fig 7A). An in-gel staining assay for NADH dehydrogenase activity gave a consistent result (Fig 7B). Similarly, assays targeting F_1_F_o_-ATPase hydrolysis activity and assembly showed that neither F_1_F_o_-ATPase nor the free F’ and F_1_ moieties were formed in the mutant (Fig 7C and 7D), indicating that the assembly and activity of complex V were both compromised by the loss of *Emp21* function. In contrast, the abundance of complex III was markedly increased in the mutant (Fig 7E). The outcome of a series of Western blot experiments was that the abundance in the mutant of Nad9 (complex I) was greatly reduced, that of Atp1 (complex V) was barely detectable, that of Cox2 (complex IV) was unaffected and that of Cytc1 (complex III) was greatly increased (Fig 7F). Thus, the loss of EMP21 function clearly impaired the assembly and function of mitochondrial complexes I and V. An up-regulation on complex III was probably the result of the regulatory mechanism of the complex gene expression.

The block of the cytochrome pathway of the respiratory chain often leads to enhanced alternative pathway [24,61,66,67]. Three *ZmAOX* genes (*ZmAOX1*, *ZmAOX2* and *ZmAOX3*) were found in the maize genome [68]. Both RT-PCR and qRT-PCR assays indicated that the abundance of *ZmAOX2* and *ZmAOX3* transcripts was much higher in the *emp21-1* mutant than in wild type (S8A and S8B Fig), while, consistently, the measured abundance of AOX protein was increased (Fig 7F). Together, these results indicate that EMP21 is crucial for the biogenesis and activity of complexes I and V in maize mitochondria.

### Genetic analysis of *emp21* and *emp*5

**Fig 8:**
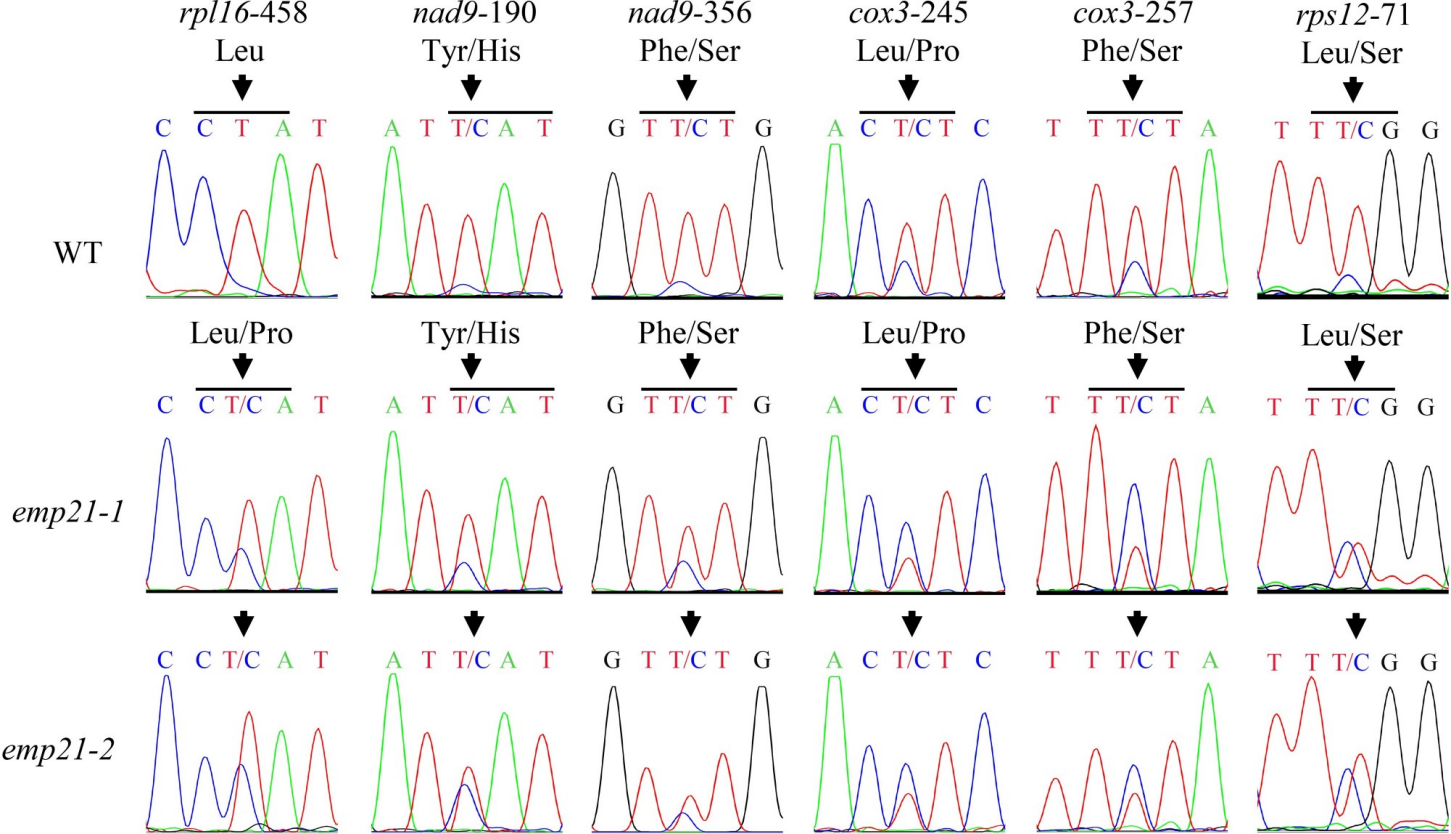
The overlapped RNA editing sites in *emp21* and *emp5-1*. Editing profile of these six overlapped RNA editing sites in *emp21*. Sites subject to defective editing marked by arrows. The residues shown on the left are generated by an edited codon and those on the right by a non-edited codon.

**Fig 9:**
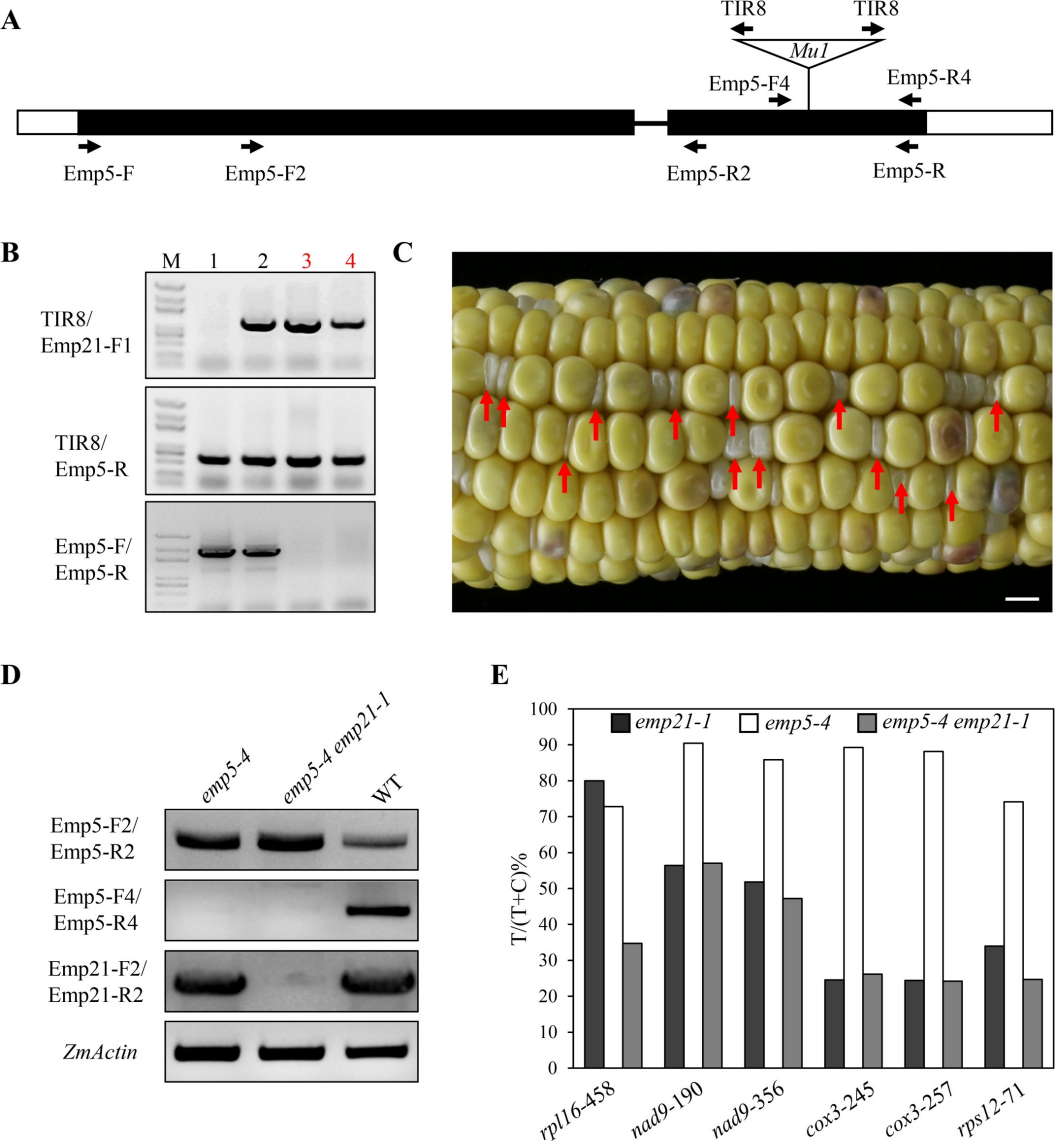
The enhanced effect on the editing of *rpl16*-*458* within the *emp5-4 emp21-1* double mutant. (A) Gene structure of *Emp5*. Protein coding region is in black and the *Mu1* insertion is indicated by a triangle. (B) PCR-based identification of *emp5-4/emp5-4 Emp21/emp21-1* plants. The lane 3 and 4 marked by red font are *emp5-4/emp5-4 Emp21/emp21-1* plants. (C) A selfed ear of the *emp5-4/emp5-4 Emp21/emp21-1* double mutant. The *emp5-4 emp21-1* double mutant kernels are marked by red arrows. Bar = 1 cm. (D) Transcription profiling of *Emp5* and *Emp21* in the *emp5-4* single mutant, the *emp5-4 emp21-1* double mutant and WT. (E) Editing efficiency in the *emp21-1* and *emp5-4* single mutants and the *emp5-4 emp21-1* double mutant. The relevant raw data are reported in S4 Dataset.

**Fig 10:**
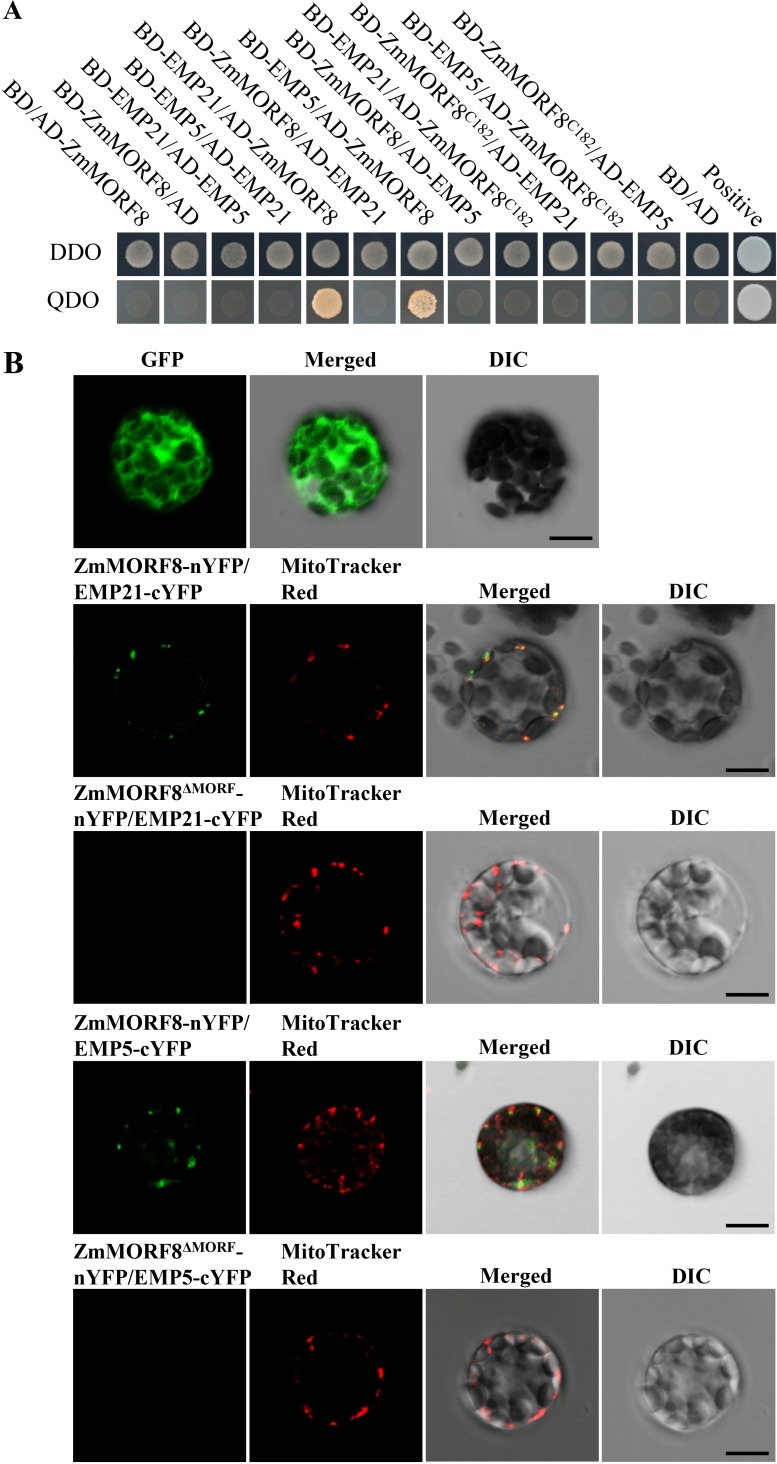
EMP21 and EMP5 interact with ZmMORF8. **(A)** A yeast two hybrid (Y2H) assay identifies interactions among EMP21, EMP5 and ZmMORF8. The colony pictures were taken after three days incubation at 30°C in SD/-Trp-Leu dropout (DDO) plates, as well as six days incubation at 30°C in SD/-Trp-Leu-His-Ade dropout (QDO) plates. (B) A BIFC assay identifies interactions between EMP21 and ZmMORF8, as well as between EMP5 and ZmMORF8. Florescence signals observed by laser confocal microscopy ZEISS LSM 880. Mitochondria stained using MitoTracker Red. DIC: differential interference contrast. Bar = 10 μm.

EMP5 is found to be required for the editing of 10 sites in maize mitochondrial transcripts [18], and six of these sites overlap with those of EMP21 (Figs 5B and 8 and S2 Dataset). The C-to-U editing of *rpl16*-458 was less effective in the *emp21-1* mutant than in wild type (Fig 8 and S2 Dataset), while it is abolished in the *emp5-1* mutant [18]. In addition, the editing of *nad9*-190, *nad9*-356, *cox3*-245, *cox3*-257, and *rps12*-71 sites was reduced in both *emp21* and *emp5-1* (Fig 8 and S2 Dataset) [18]. In the *emp5-4* allele, mutational *Emp5-4* encodes a truncated EMP5 protein lacking the E+ and DYW domains. Most of the editing events affected by EMP5 show similar editing levels in *emp5-4* and wild type, except the editing of *rpl16*-458 which is decreased compared with wild type [18], promoting the idea that the EMP5-4 mutant protein without the DYW domain may interact with another PPR-DYW protein to facilitate editing. To explore the genetic relationship between *Emp21* and *Emp5*, we generated double mutants from the cross *Emp21*/*emp21-1* x *Emp5*/*emp5-4*. The *emp5-4*/*emp5-4 emp21-1*/*Emp21* plants were identified by PCR in F_2_ (Fig 9B). Kernels in these double mutant selfed ears exhibited 1:2.8 segregation ratio of emp to normal kernels (Fig 9C), where the normal kernels proved to be *emp5-4* single mutants and the empty pericarp ones are *emp5-4 emp21-1* double mutants (Fig 9C and 9D). An analysis of the editing profile at the six shared sites in the double and single mutants using both STS-PCRseq and direct sequencing, showed that only 35% of the *rpl16*-*458* sites were edited in the *emp5-4 emp21-1* double mutant, as against 73% in the *emp5-4* single mutant and 80% in the *emp21-1* single mutant (Figs 9E and S9 and S2 and S4 Dataset). The editing efficiency of *nad9*-190, -356, *cox3*-245, -257, and *rps12*-71 sites in the *emp5-4 emp21-1* double mutant was similar to that in the *emp21-1* single mutant (Fig 9E). Because the editing of *rpl16*-458 site is completely dependent on the presence of EMP5, these results suggested that a portion (~30%) of the *rpl16*-458 sites are edited by EMP21 and EMP5 jointly. To determine whether EMP21 directly interacts with EMP5, a yeast two hybrid (Y2H) assay was conducted. The yeast cells containing BD-EMP21/AD-EMP5 set or BD-EMP5/AD-EMP21 set did not grow on the SD/-Trp-Leu-His-Ade dropout plates (Fig 10A), suggesting that these two proteins may not interact in the yeast.

### EMP21 and EMP5 can interact with ZmMORF8

MORFs/RIPs are responsible for the editing at most of the sites in the mitochondrial and plastidial transcripts in Arabidopsis [37,40,59]. Functions of the maize MORFs/RIPs are not identified. We analyzed the 81 EMP21 edited sites with respect to the sites edited by MORFs/RIPs in Arabidopsis. Interestingly, 44 of the 81 sites edited by EMP21 in maize do not need editing in Arabidopsis as these sites are mostly “Ts” (S2 Table). Among the rest 37 edited sites, 34 require the editing function of MORF8 in Arabidopsis (S2 Table). Eight editing sites mediated by MORF8 overlap with those mediated by EMP5 (S2 Table). A BLAST search identified six putative mitochondrion-targeted MORF orthologs in maize, named ZmMORF1 (GRMZM2G003765), ZmMORF3 (GRMZM2G054537), ZmMORF4 (GRMZM2G139441), ZmMORF5 (GRMZM2G383540), ZmMORF6 (GRMZM5G808811) and ZmMORF8 (GRMZM2G169384). The overlaps promoted us to explore the relationship among EMP21, EMP5 and six ZmMORFs. The results of Y2H assays indicated that both BD-EMP21 and BD-EMP5 are able to interact with AD-ZmMORF8, but not with other five ZmMORFs (Figs 10A, S10 and S11). However, the reciprocal mating pairs did not grow on the SD/-Trp-Leu-His-Ade dropout plates (Fig 10A). It is possible that BD-ZmMORF8 or AD-EMP21/AD-EMP5 cannot be properly expressed in yeast. Deletion of the MORF domain in ZmMORF8^C182^ abolished the interaction with EMP5 and EMP21 (Figs 10A and S12). ZmMORF4 displayed auto-activation when fused to the BD domain, hence tested in AD-ZmMORF4 (S10 Fig). The implied direct interactions between ZmMORF8 and both EMP21 and EMP5 were further verified using a bimolecular fluorescence complementation (BIFC) assay. After co-expressing the N-terminal YFP fusion of ZmMORF8 and C-terminal YFP fusion with either EMP21 or EMP5 in Arabidopsis protoplasts, we observed the punctated dot YFP signals merged with the mitochondria which were stained by the MitoTracker Red (Fig 10B). No signal was generated in protoplasts co-expressing a fusion between the YFP N terminus and a truncated version of ZmMORF8^ΔMORF^ with deleted MORF motif, and C-terminal YFP fusion with EMP21 or EMP5 (Figs 10B and S12). When the truncated EMP21 and EMP5 (either only the PPR motifs, or the PPR+E motifs, or the E+DYW motifs) were tested, a weak interaction was observed between ZmMORF8 and both the PPR motifs and the PPR+E motifs, while the E+DYW motifs failed to interact (S11 Fig). These results suggest that EMP21 and EMP5 function in the editing at some sites by interacting with ZmMORF8 and this interaction depends mainly on the PPR motifs of these two PPRs.

## Discussion

This study revealed a novel PPR-DYW protein that is required for the C-to-U editing at about 17% target Cs in mitochondrial transcripts. EMP21 is essential to the editing of *nad7*-77, *atp1*-1292, *atp8*-437, *nad3*-275 and *rps4*-870, and additionally required for the editing of 76 sites in 21 transcripts. A failure to edit at most of these sites resulted in an altered translation product, with potential consequences for the gene product’s functionality. In particular, the absence of EMP21 resulted in an impaired assembly, and consequently a reduced level of activity of mitochondrial complexes I and V, with knock-on effects on embryogenesis and endosperm development. Interestingly, there is some commonality with respect to the editing sites targeted by EMP21 and EMP5 [18], implying that effective editing at these sites requires the presence of both proteins. However, Y2H assays did not detect any interaction between the two proteins, rather found that EMP21 and EMP5 can directly interact with ZmMORF8. Thus, the editing of some sites may involve interaction between EMP21 and ZmMORF8 *in vivo*.

### Defective editing results in dysfunctional mitochondria and compromised kernel development

Experimental evidence has shown that abolishing editing in mitochondrial genes can disturb mitochondrial functionality and thereby inhibit the development of the maize kernel [18,24,67,69,70]. For example, an analysis based on the behavior of mutants has demonstrated that the *rpl16*-458 site requires EMP5 (a DYW-subclass PPR protein) to perform the editing needed to support normal kernel development [18]. Similarly, in the *emp9* mutant, editing at *ccmB*-43 is abolished, resulting in the translation of a Pro rather than a Ser residue; this single residue change is sufficient to disrupt the assembly of complex III and results in kernel abortion [24]. Meanwhile, the effect of mutating *Emp18*, which encodes a mitochondrial PPR-DYW protein involved in editing the *atp6-*635 site, is to convert a Leu to Pro in Atp6, a subunit of F_1_F_o_-ATPase; the alteration disrupts the α-helix of subunit a, leading to the disassembly and reduced activity of complex V, finally resulting in embryo lethality and a failure in endosperm development [69]. In the present *emp21* mutants, the non-editing of *atp1*-1292 resulted in the translated Atp1 protein carrying a Pro rather than a Leu at position 431 (Fig 5A). Atp1 is the α-subunit in F_1_-factor of complex V (F_1_F_o_-ATPase), a multimeric enzyme (α3β3γδϵ) in mitochondrial respiratory chain [71]. Based on the structure of its ortholog [72], Leu^431^ lies within the conserved α-helix, so the failure to correct this residue probably disrupts the α-helix, so likely compromising the assembly of complex V (Fig 7C and 7D). The *emp21* mutation also abolished editing at *atp8*-437 and reduced its effectiveness at *atp8*-436 (Fig 5A). As this residue is located in the non-conserved C terminal region of Atp8 (Fig 6C), this argues a possibility that the mutated forms of Atp8 have a (moderate) impact on complex V. Considering the conservation of the editing site at *atp1-*1292, as well as the lack of complex V assembly and activity, the editing deficiency of *atp1-*1292 probably causes the defective complex V and arrested embryogenesis and endosperm development in *emp21*.

In addition to its influence over editing at *atp1-*1292 and *atp8-*437, the loss-of-function of *Emp21* also abolished the editing at *nad7-*77 and *nad3-*275, as well as resulting in a reduction in the effectiveness of editing at one site in *nad2*, 16 in *nad3*, one in *nad4*, six in *nad6* and two in *nad9* (Figs 5A and S6 and S2 Dataset)–these genes all encode subunits of mitochondrial complex I. In the mutant, the effect at *nad7*-77 resulted in a change from the wild type residue at position 26 (Leu) to Ser (Fig 5A). The Leu^26^ residue is widely conserved across both higher and lower plants (Fig 6A). In the porcine accessory subunit NADH dehydrogenase iron-sulfur protein 2, which shares 69.6% identity with Nad7 [73], the Leu^26^ residue is located in the highly conserved AHGVLR linker between two β-sheets. A Leu^26^ to Ser^26^ change probably disrupts the protein stability, as suggested by the behavior of maize *dek36* mutants. An E+-subgroup PPR DEK36 being responsible for *nad7*-383 editing, converts Ser to Leu located in a highly conserved VGALT linker between two α-helixes in Nad7. Mutation of DEK36 dramatically impairs the stability of Nad7 and activity of complex I [60]. Defective editing at multiple sites in the *nad3* transcript may similarly have contributed to the observed inhibition of complex I assembly and activity noted in the *emp21* mutant. Such as, the editing at *nad3*-247 (*nad3*-250 in Arabidopsis) is severely decreased in *emp21* (S6 Fig and S2 Dataset). Defective editing at *nad3*-250 has been implicated as strongly impairing the complex I activity in the Arabidopsis *slg1* mutant [74]. The effectiveness of editing at a further 50 sites, scattered across 17 transcripts, was reduced in *emp21* (S6 Fig and S2 Dataset), but this seems unlikely to have contribution to mitochondrial dysfunction, since complex III assembly was enhanced in the *emp21-1* mutant (Fig 7E), and the abundance of other respiratory chain proteins (notably Cox2) was indistinguishable from that present in wild type (Fig 7F). The conclusion is that the compromised kernel development induced in the *emp21* mutants is likely attributable to a failure to convert cytidine to uridine at *atp1*-1292 and *nad7*-77, in conjunction with a reduced level of conversion at multiple *nad3* sites.

### EMP21 is novel PPR-DYW protein that is required for the editing of *~*17% target Cs in maize mitochondria

Many PLS-class PPRs have been identified as factors involved in the C-to-U editing in mitochondria and plastids; roughly half belong to the DYW-subclass (S3 Table). Most of these proteins each target only a small number of sites for editing, the exceptions being DYW2 [75,76], NUWA [75], EMP21 (this study) and MEF8 [62] which target, respectively, 392, 223, 81 and 38 sites. Both DYW2 and MEF8 are atypical DYW-subclass proteins lacking a canonical E domain and harbor only five PPR repeats which are thought not sufficient to confer a tight specificity on the substrates [62,75]. And DYW2 functions in both plastids and mitochondria. NUWA is a P-class of PPR protein lacking the DYW and E domain which is usually not found to have the editing function. NUWA is also targeted to mitochondria and plastids [75,76]. In this context, EMP21 is novel among these proteins in which it is a canonical PPR-DYW protein possessing conserved E and DYW domains and eleven PPR-motifs (Fig 4A).

The requirement of EMP21 and the above three other PPR proteins required for the editing of such a large number of sites provides certain clues to the editing machinery in plant organelles. PPR proteins are thought to bind to the upstream sequences of the target Cs by one PPR-repeat one nucleotide manner based on the amino acid at the 6 and 1’ position of the PPR repeats [63–65]. Such binding has been verified in several reports [33,77]. In consistent with this binding codes, we found a good agreement between the EMP21 repeats and the upstream sequences of *nad7*-77, *atp1*-1292 and *atp8*-437 (S1 Table). EMP21 is essential to the editing of those three sites. However, the upstream sequences of other 78 editing sites are not aligned well with the EMP21 repeats (S1 Table). We considered the possibility that the defective editing in the *emp21* mutant at these 78 sites represents a secondary effect caused by compromised mitochondrial function, but this explanation is not supported by the behavior of other mutants. One such example is the *smk1* mutant which features a severely reduced assembly and activity of complex I and abnormal mitochondria. However, SMK1 only functions in the editing of *nad7*-836, and no other editing sites are affected in the *smk1* mutant [67]. A second example relates to EMP18 in maize; when *Emp18* is disabled, editing is only compromised at two sites [69]. More generally, it is well established that defective editing of mitochondrial transcripts is not an inevitable consequence of mitochondrial dysfunction [24,66,70,78]. Thus, the dysfunctional mitochondria cannot result in the decreased editing of these sites in *emp21*. An alternative possibility is that these 78 sites are not specified by EMP21, but rather by other PLS-class PPRs that exist in an editosome where the DYW domain of EMP21 containing the conserved CDAs-like signature residues (HxE(x)nCxxC) (S5 Fig) provides the deaminase activity. This hypothesis is supported by the finding that DYW domains are the cytidine deaminase operating on RNA editing [27]. It is also in agreement with the finding that roughly half of the editing sites recognition cannot be explained by the one PPR-repeat one nucleotide codes in the PPR-DYW and PPR-E proteins [65]. Accumulating evidence points to the likelihood that editing is carried out by large ribonucleoprotein complexes composed of a variety of PLS-PPRs, MORFs/RIPs, ORRMs, OZ1, certain P-subclass PPRs and other proteins in flowering plant [55,75,76,79]. The DYW2 protein has been proposed to be recruited to specific sites by E+-subclass PPRs, where it provides the necessary deaminase activity; meanwhile NUWA supports the interaction between the E+-subclass PPRs and DYW2 [75,76]. In addition, an *in vivo* pull-down assay has demonstrated that MORF1 connects with DYW2 and NUWA [80]. These are clear evidence that editing involves a large complex that mainly serve to recruit functional DYW domains by (multiple) protein-protein interaction.

### The editing of certain sites by EMP21 and EMP5 involves interactions with ZmMORF8

We have uncovered that EMP21 and EMP5 are required for the editing of six overlapping sites in mitochondria (Figs 5B and 8) [18]. The *emp5-4* allele, which shows reduced editing of *rpl16*-458, may be able to encode a truncated product lacking the DYW domain but retaining the E domain. It is proposed that this truncated protein may still possess the editing function by recruiting other PPR-DYW proteins [18]. The *emp5-4 emp21-1* double mutant displayed substantially reduced editing efficiency at *rpl16*-458 (Figs 9E and S9): the editing efficiency at this site in the single mutants was approximately 73% (*emp5-4*) and 80% (*emp21-1*), falling to 35% in the double mutant. Both the Y2H and BIFC assay confirmed that EMP5 and EMP21 interacted with ZmMORF8 (Fig 10A and 10B). Since EMP5 is essential for the editing at *rpl16*-458 site [18], it is possible that EMP5 specifies the *rpl16*-458 site and recruits either ZmMORF8 and/or EMP21 (or possibly other PPRs) to enable the editing process. This provides a reasonable explanation that loss of the DYW domain of EMP5 can be partially complemented by EMP21 and the C-to-U editing is carried out by protein complexes. The Y2H assay implied that EMP5 did not directly interact with EMP21 (Fig 10A). As reported elsewhere, the two PPR-E+ proteins CLB19 and SLO2 showed either no, or at best a weak direct interaction with DYW2, while a P-type PPR NUWA, detected as PPR- E+-interacting partner, bridges and stabilizes the interaction between PPR-E+ and the DYW protein [75,76]. Thus, it is possible that an as yet unidentified P-type PPR protein (or perhaps some other editing factor(s)) are needed to support an interaction between EMP5 and EMP21.

### Increased editing at some sites in the *emp21* mutant

The loss-of-function of *Emp21* caused an increase in the editing at 22 sites in 11 transcripts (Figs 5C and S7 and S2 Dataset). This phenomenon has also been reported in the Arabidopsis *dyw2*, *mef8* and *reme1* mutants, as well as the maize *emp5* mutant [18,62,75,76,81]. Absence of DYW2, an atypical PPR protein, decreased the editing efficiency of over 300 sites while increased the editing of over 90 sites [75]. Null mutation of MEF8, another atypical PPR protein, exhibited reduced editing at 38 sites and increased editing at 24 sites [62]. REME1 is a typical PPR-DYW, and its absence decreased in the editing extent of two sites and increased in the editing extent of two sites [81]. In maize, the loss of the functional EMP5 (a typical DYW-type PPR) resulted in a decrease at 10 sites, along with an enhancement in editing effectiveness at 5 sites [18]. It appears that in these mutants the more sites decreased in editing is associated with the more sites increased in editing, and that the increase in editing has site-specificity. For example, the editing efficiency at the *mttB*-552 and *nad2*-558 sites is reduced in *reme1* [81], whereas in *dyw2*, the editing efficiency is reduced at the *mttB*-552 site, but enhanced at the *nad2*-558 site [75,76]. As enhanced editing results from increased expression of editing factors (in editosome) mostly encoded by the nuclear genes, signal transduction is expected to be involved between the nucleus and mitochondrion. Dysfunction of mitochondria may trigger this signal transduction from mitochondria to nucleus which selectively up-regulate the expression of genes with function in mitochondria. Which signals and how these genes are regulated remain to be elucidated. This provides a possibility that the impaired processes in *emp21* mitochondria enhance the expression of certain editing factors, which results in increased editing at certain sites. Another possibility is that absence of one editing factor leads to increased formation of other editing complexes. This possibility lays on an assumption that components in editosomes are highly dynamic and in equilibrium. It is equally possible that some PPR-DYW proteins play an inhibitory role on mitochondrial editing [62]. This hypothesis is supported by the phenomenon that approximately 75% of the sites with increased editing efficiency in the *mef8* mutant returned to almost normal level when complemented by a mutated MEF8 (HxE→HxA in DYW domain) [62]. The nature of this inhibition remains a question, but this hypothesis is consistent with the notion that components of editosome are highly dynamic and in equilibrium, as the mutated MEF8 can still be incorporated in the complexes.

## Methods

### Plant materials and growth conditions

The *emp21* alleles which render nearly isogenic W22 background (99.6%) were isolated from the UniformMu mutagenic population [56]. Maize (*Zea mays*) was grown in the experimental field at Shandong University in Jinan, Shandong province under natural conditions. Wild type and transgenic Arabidopsis were grown at 22°C with 16 h light and 8 h dark in culture room.

### Light microscopy of cytological sections

Wild type and mutant kernels were harvested at multiple developmental stages (12 and 16 DAP) from selfed ears in the *emp21* heterozygous plants. The kernels were cut along longitudinal axis, and the slices containing embryo and endosperm were fixed in 4% paraformaldehyde at 4°C for 24 h. After dehydration in an ethanol gradient series (50, 70, 85, 95, and 100% ethanol), the materials were cleared with xylene and infiltrated by paraffin wax. And then, the samples were embedded in paraffin wax and sectioned at 10 μm thickness by using the Leica 2035 Biocut. The sections were stained with Johansen’s Safranin O and observed with ZEISS microscope.

### DNA extraction and gene cloning

Genomic DNA was isolated by a urea-phenol-chloroform-based method [82]. 0.1 g fresh leaf tissues were broken by bead grinding and resuspended with 500 μl of DNA extraction buffer (7 M urea, 0.3 M NaCl, 50 mM Tris-HCl, 24 mM EDTA, and 1% sarkosine, pH 8.0). After mixing with chlorofrom-isoamyl alcohol (25:24:1), the mixture was gently shaked for 30 min at room temperature, and then separated by centrifugation at 14000 rpm for 15 min. The supernatant was transferred into a new 1.5 ml tube and mixed with 0.1 volume of 3 M sodium acetate (PH 5.2) and 380 μl isopropanol. DNA was pelleted at 14000 rpm for 15 min, washed with 70% ethanol two times, and dissolved in TE buffer (10 mM Tris-HCl, 1 mM EDTA, pH 8.0). The *Mutator* (*Mu*) insertion flanking sequences were identified by *Mu*-seq strategy as described previously [57].

### Subcellular localization

To investigate the localization of EMP21, the full-length (without stop codon) and the truncated gene fragments encoding 511 amino acids peptide at N-terminus were amplified from W22 genomic DNA and cloned into pENTR/D-TOPO (ThermoFisher Scientific, http://www.thermofisher.com). And then, *pGWB5-Emp21* or *pGWB5-Emp21*^*N511*^ vectors, which express EMP21-GFP and EMP21^N511^-GFP fusion protein, respectively, were constructed by Gateway site-specific recombination. These vectors were transformed into *Agrobacterium tumefaciens* strain EHA105. The strains carrying *pGWB5-Emp21* and *pGWB5-Emp21*^*N511*^ vectors were used to transform Arabidopsis Columbia-0 by the floral-dip [83]. The transgenic plants were screened in MS medium containing hygromycin and identified by PCR using primers GFP-R and EMP21-F2. The protoplasts were isolated from transgenic plants using described method [18], and detected by ZEISS LSM 880 confocal microscope. The mitochondria were stained by MitoTracker Red (ThermoFisher Scientific).

### RNA extraction, RT-PCR and qRT-PCR

Total RNA was extracted from wild type and *emp21* embryo and endosperm at 12 DAP using the TRIzol reagent (ThermoFisher Scientific, www.thermofisher.com) and was treated with DNase I (New England Biolabs, www.neb.sg) to remove any contaminating genomic DNA. Single-stranded cDNA was generated from the RNA via a reverse transcription reaction primed with random hexamers, using a Transcriptor First Strand cDNA Synthesis kit (ThermoFisher Scientific). Quantitative real-time polymerase chain reaction (qRT-PCR) was carried using LightCycler 96 (Roche Diagnostics). The relative gene expression value was calculated with the 2^^(-ΔΔCt)^ fomular. The expression level of *ZmActin* (GRMZM2G126010) served as the reference to normalize the target gene expression. And each experiment was replicated three times. The primers used by RT-PCR and qRT-PCR were shown in S4 Table.

### RNA editing analysis through STS-PCRseq

The STS-PCRseq [59] method was applied to characterize RNA editing in the maize kernel. Embryo and endosperm tissue from kernels sampled at 12 DAP was prepared from both wild type (WT) and *emp21* kernels (WT-1 and *emp21-1*, WT-2 and *emp21-2*) set by plants heterozygous for the respective mutant allele. The 35 targeted mitochondrial genes were PCR-amplified from the cDNA templates obtained as described above (primers given in S4 Table). The RT-PCR amplicons obtained from each template were mixed in an equimolar ratio and sheared by sonication. Sequencing libraries were generated using a NEB Next Ultra DNA. Library Prep kit for Illumina (New England Biolabs) following the manufacturer’s protocol and index codes were added in order to allow sequences to be attributable to each sample. The quality of each library was assessed using a Bioanalyzer 2100 system device (Agilent). The four resulting DNA libraries were sequenced using a Hiseq Xten-PE150 instrument. Read quality control, read trimming and alignment were performed following the SNP-calling method given in [84]. The threshold for declaring a difference in editing effectiveness was defined as: (T/(T+C)% in *emp21*-T/(T+C)% in WT) had to be ≤-10% (decrease of editing in *emp21*) or ≥10% (increase of editing in *emp21*) for all the four pairwise comparisons (*emp21-1* vs. WT-1, *emp21-1* vs. WT-2, *emp21-2* vs. WT-1, *emp21-2* vs. WT-2). This method was modified from the previously reported [62]. The same method was used to detect the extent of editing at six sites in the *emp5-4* mutant and the *emp5-4 emp21-1* double mutant.

### Direct sequencing of RT-PCR amplicons

Embryo and endosperm samples were dissected from wild type and *emp21* kernels at 12 DAP sampled from three independent ears. An RNA editing analysis was conducted from these samples by directly sequencing the RT-PCR amplicons, as described in [18]. The necessary cDNA was obtained as described above and subjected to a series of RT-PCRs directed at the full set of 35 mitochondrial genes (primers given in S4 Table). And each experiment was replicated three times.

### Blue Native-PAGE and complexes I and V activity assay

Mitochondria were isolated from embryo and endosperm of *emp21-1* and wild type at 12 DAP. The Blue native polyacrylamide gel electrophoresis (BN-PAGE) and in-gel complex I activity analyses were performed as previous report [85]. The complex V activity assay was performed following the description by Wittig et al [86].

### Western blotting

Mitochondrial proteins extracted from embryo and endosperm at 12 DAP were separated by BN-PAGE and/or SDS-PAGE. And then proteins were transferred to the nitrocellulose membrane. Proteins were detected by using specific antibodies as described previously [66].

### Yeast two hybrid assays

The Y2H assays were performed as described previously by Glass et al. 2015 [41]. Briefly, the fragments (without the targeted peptide) of *Emp21*, *Emp5*, and *ZmMORFs* and truncated fragments of *Emp5*, *Emp21* and *ZmMORF8* (*ZmMORF8*^*C182*^, S12 Fig) were recombined into either the GAL4 activation domain plasmids (*pGADT7*) or the GAL4 binding domain plasmids (*pGBKT7*) using restriction enzyme ligation (Clontech Laboratories). Both plasmids were then co-transformed into yeast strain Y2H Gold. Protein–protein interactions were determined by measuring in SD/-Trp-Leu-His-Ade dropout (QDO) and SD/-Trp-Leu-His-Ade dropout + x-α-gal (QDO+ x-α-gal) plates for 6 days at 30°C.

### Bimolecular Fluorescence complementation assay

To investigate the interaction among ZmMORF8, EMP21 and EMP5, plasmids containing N- and C-terminal fusions of YFP were co-transformed into Arabidopsis protoplasts as previously described [87]. The *ZmMORF8*^*ΔMORF*^ (S12 Fig) which was deleted MORF box was cloned by fusion PCR using primers ZmMORF8-F14/F19 and ZmMORF8-R14/R19 (S4 Table). The protoplasts were observed using ZEISS LSM 880 after incubating under dark for 24–30 h. The mitochondria were stained by MitoTracker Red (ThermoFisher Scientific).

## Supporting information

S1 Dataset Number of reads at each editing site (gene-position) for each library. (XLSX)

S2 Dataset The editing sites in maize mitochondria and the affected editing sites in *emp21*. (XLSX)

S3 Dataset The editing extent of the 493 mitochondrial editing sites and the amino acid residues encoded by the editing sites. (XLSX)

S4 Dataset Number of reads covering the six overlapping editing sites in the *emp5-4* single mutant and the *emp5-4 emp21-1* double mutant. (XLSX)

S1 Fig Linkage analysis of *emp21-1*. (A) The position of the *Mu* insertion site in *Emp21* (marked by a triangle) and the positions of the primers used for genotyping. (B) Linkage analysis in an F_2_ population segregating *emp21-1*. The 1153 bp band amplified by PCR using Emp21-F1/TIR8 primers is derived of a *Mu* insertion in the *Emp21* gene. The 2001 bp band amplified by PCR using Emp21-F1/Emp21-R1 primers indicates the wild type *Emp21*. N, non-segregating; S, segregating.
(TIF)

S2 Fig The allelism analysis of the *emp21* mutants. (A) Selfed ear of an *emp21-2* heterozygote. (B, C) Allelism test using the reciprocal crosses (B) *Emp21/emp21-1* × *Emp21/emp21-2*, (C) *Emp21/emp21-2 × Emp21/emp21-1*. Empty pericarp kernels are marked by red arrows. Bar = 1 cm.
(TIF)

S3 Fig Conserved residues in the PPRs and the E1 and E2 motifs of EMP21 based on previous publication [8]. Highly conserved residues are shown shaded.
(TIF)

S4 Fig Alignment of the maize EMP21 with putative orthologs from sorghum (SbEMP21), wheat (TaEMP21) and rice (OsEMP21). (TIF)

S5 Fig Polypeptide alignment within the DYW domain of EMP21, DYW1, DYW2, MEF8, *Pp*PPR56 and *Pp*PPR65. The conserved cytidine deaminase-like zinc binding signature residues HxE(x)nCxxC and the C terminal DYW tripeptide are shown in red font.
(TIF)

S6 Fig The decreased editing sites in *emp21*. The defective editing sites are arrowed. The residues shown on the left are generated by an edited codon and those on the right by a non-edited codon.
(TIF)

S7 Fig The increased editing sites in *emp21*. The increased editing sites are arrowed. The residues shown on the left are generated by an edited codon and those on the right by a non-edited codon.
(TIF)

S8 Fig Transcription profiling of *ZmAOX1*, *ZmAOX2* and *ZmAOX3* in wild type and *emp21-1*. RT-PCR (A) and qRT-PCR (B) analyses of *ZmAOX* genes in WT and *emp21-1*. RNA was extracted from 12 DAP embryos and endosperms. qRT-PCR values represent three biological replicates and are normalized against *ZmActin*. Error bars represent the ±SD.
(TIF)

S9 Fig Editing profiles in the *emp5-4* single mutant and the *emp5-4 emp21-1* double mutant. Defective sites are arrowed. The residues shown on the left are generated by an edited codon and those on the right by a non-edited codon.
(TIF)

S10 Fig The detection of interaction between PPRs and ZmMORFs. The colony pictures were taken after three days incubation at 30°C in SD/-Trp-Leu dropout (DDO) plates, as well as six days incubation at 30°C in SD/-Trp-Leu-His-Ade dropout (QDO) plates.
(TIF)

S11 Fig The interaction between the domains of EMP5 and EMP21, and ZmMORF8. The colony pictures were taken after three days incubation at 30°C in SD/-Trp-Leu dropout (DDO) plates, as well as six days incubation at 30°C in SD/-Trp-Leu-His-Ade dropout (QDO) plates and SD/-Trp-Leu-His-Ade dropout + x-α-gal (QDO+ x-α-gal) plates.
(TIF)

S12 Fig The protein structure of ZmMORF8, ZmMORF8^C182^ and ZmMORF8^ΔMORF^. (TIF)

S1 Table Alignment of the amino acid residues at position 6 and 1’ in each PPR motif of EMP21 with -4 to -14 bp upstream sequence of the 81 defective editing sites based on the codes previously reported [65]. The editing sites are marked by red arrows.
(XLSX)

S2 Table The affected editing sites in *emp21* and *emp5-1* and their corresponding nucleotides in Arabidopsis. (XLSX)

S3 Table Known editing PPR proteins and the defective editing sites in these mutants. (XLSX)

S4 Table Primers used for the study. (XLSX)
