# Supplementary figures and images for: Empty Pericarp21 encodes a novel PPR-DYW protein that is required for mitochondrial RNA editing at multiple sites, complexes I and V biogenesis, and seed development in maize
Source: PLoS Genet. 2019 Aug 2;15(8):e1008305. doi: 10.1371/journal.pgen.1008305 (PMC6693784; doi:10.1371/journal.pgen.1008305)

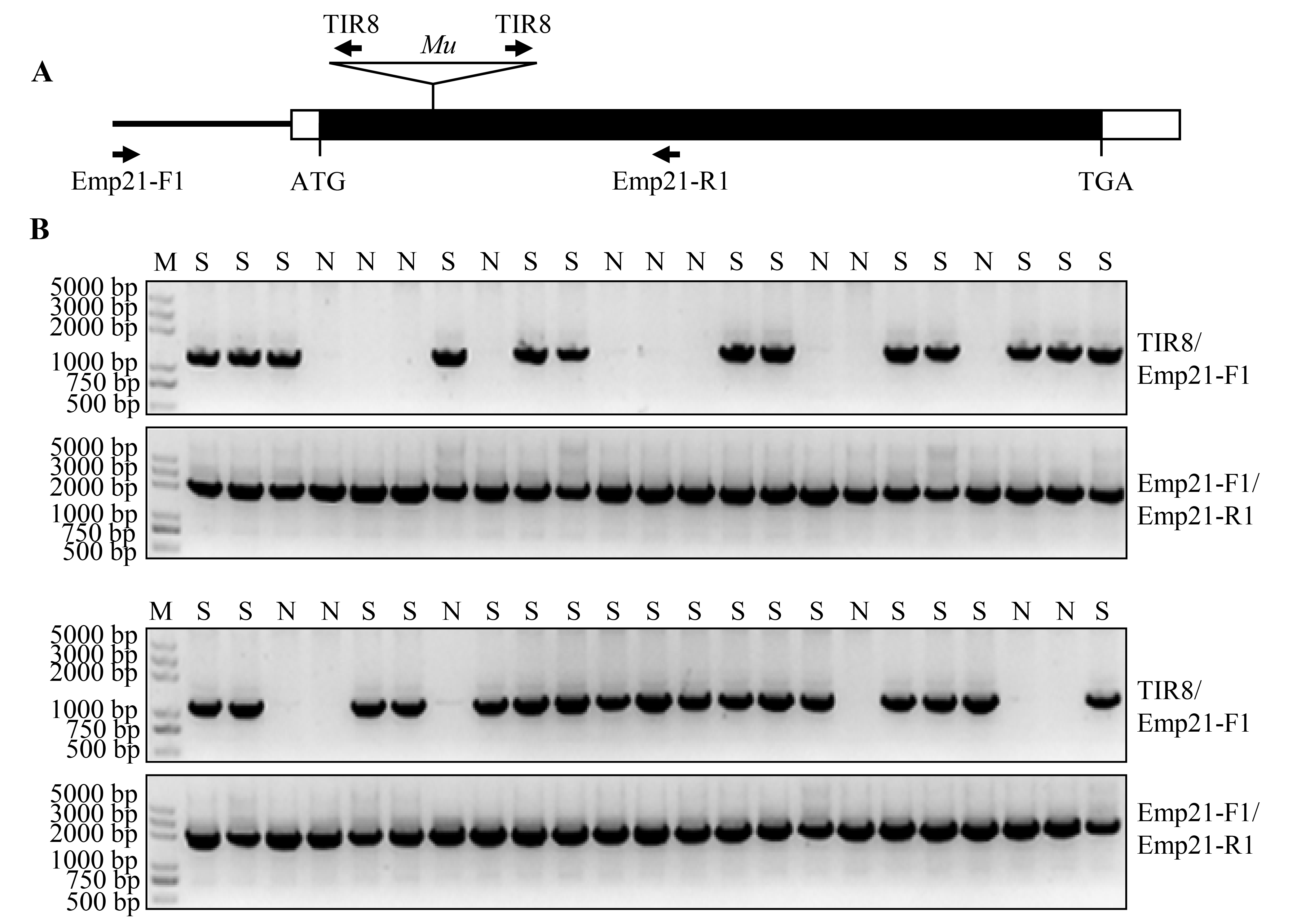

Supplement: S1 Fig — (A) The position of the Mu insertion site in Emp21 (marked by a triangle) and the positions of the primers used for genotyping. (B) Linkage analysis in an F2 population segregating emp21-1. The 1153 bp band amplified by PCR using Emp21-F1/TIR8 primers is derived of a Mu insertion in the Emp21 gene. The 2001 bp band amplified by PCR using Emp21-F1/Emp21-R1 primers indicates the wild type Emp21. N, non-segregating; S, segregating. (TIF) [file pgen.1008305.s005.tif]

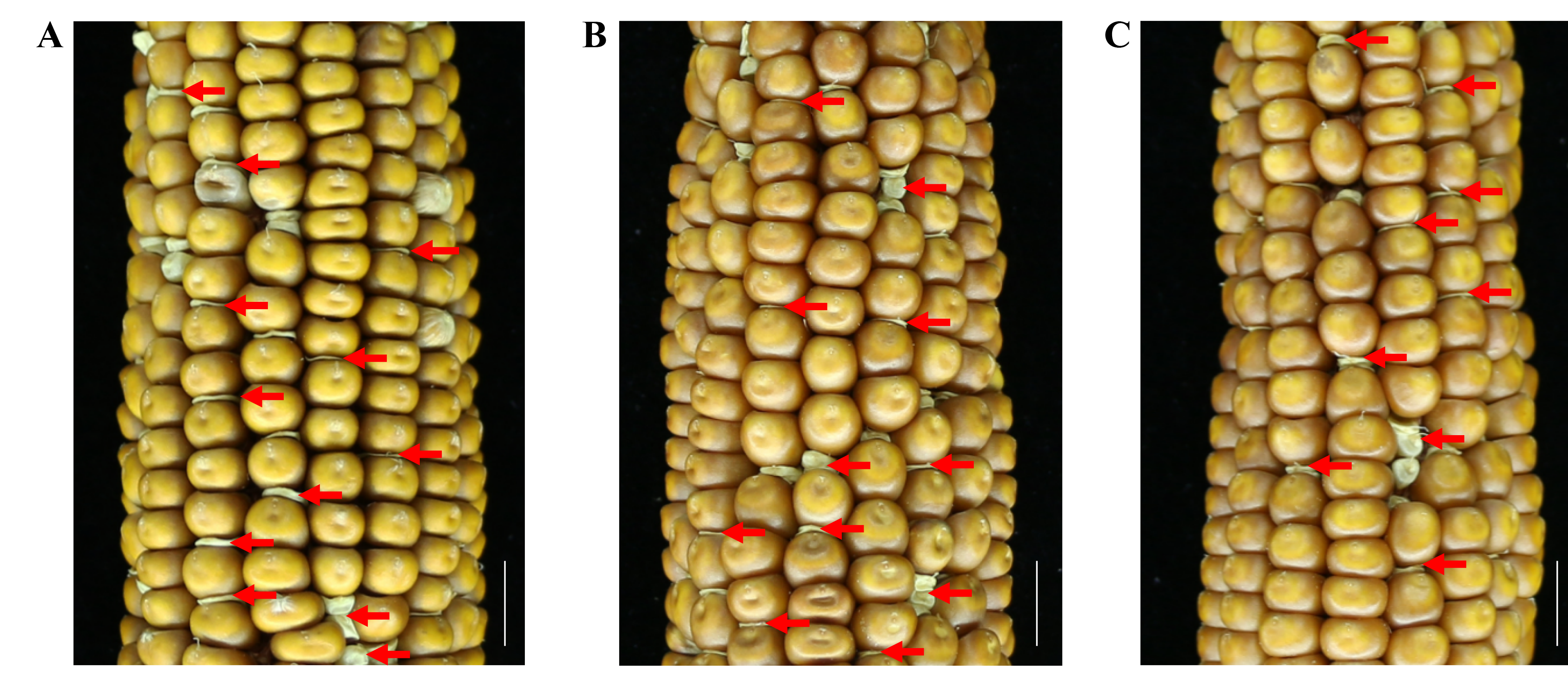

Supplement: S2 Fig — (A) Selfed ear of an emp21-2 heterozygote. (B, C) Allelism test using the reciprocal crosses (B) Emp21/emp21-1 × Emp21/emp21-2, (C) Emp21/emp21-2 × Emp21/emp21-1. Empty pericarp kernels are marked by red arrows. Bar = 1 cm. (TIF) [file pgen.1008305.s006.tif]

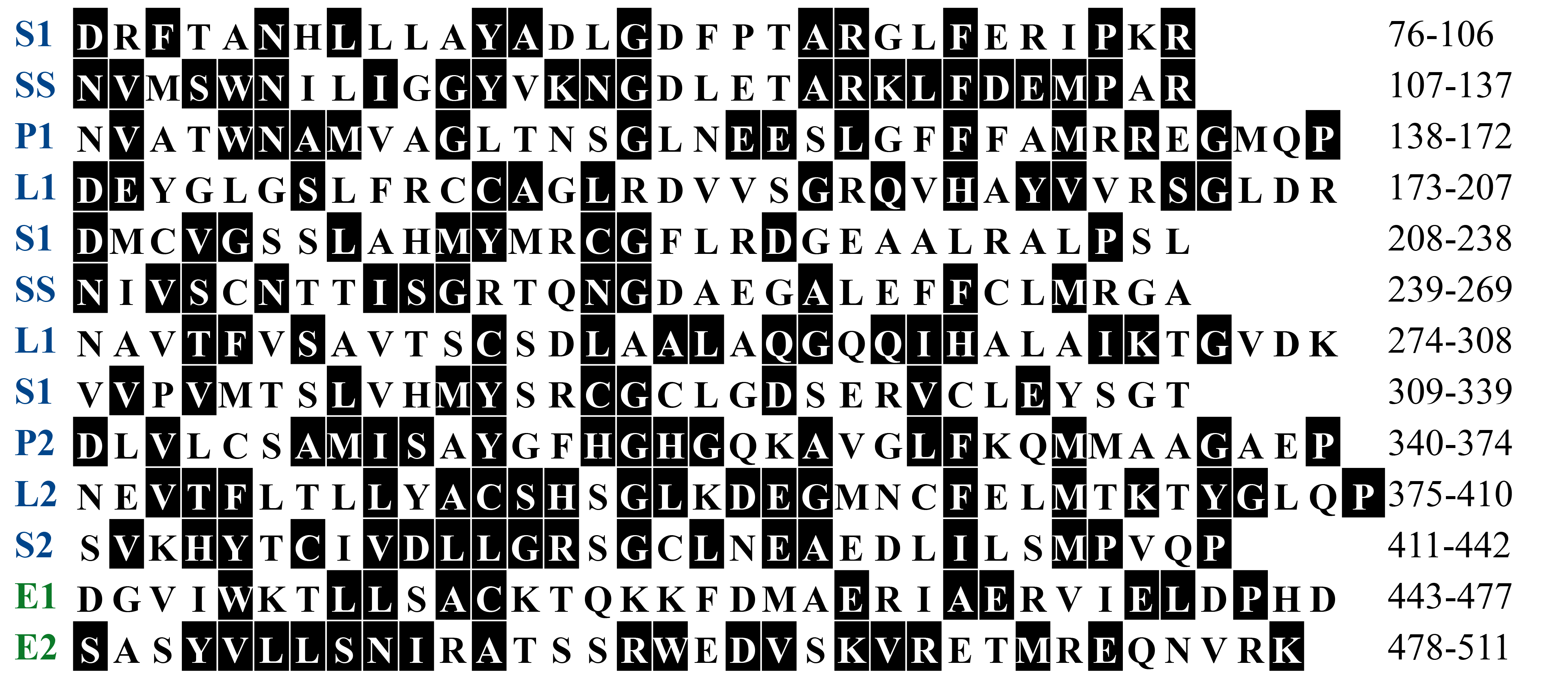

Supplement: S3 Fig — Highly conserved residues are shown shaded. (TIF) [file pgen.1008305.s007.tif]

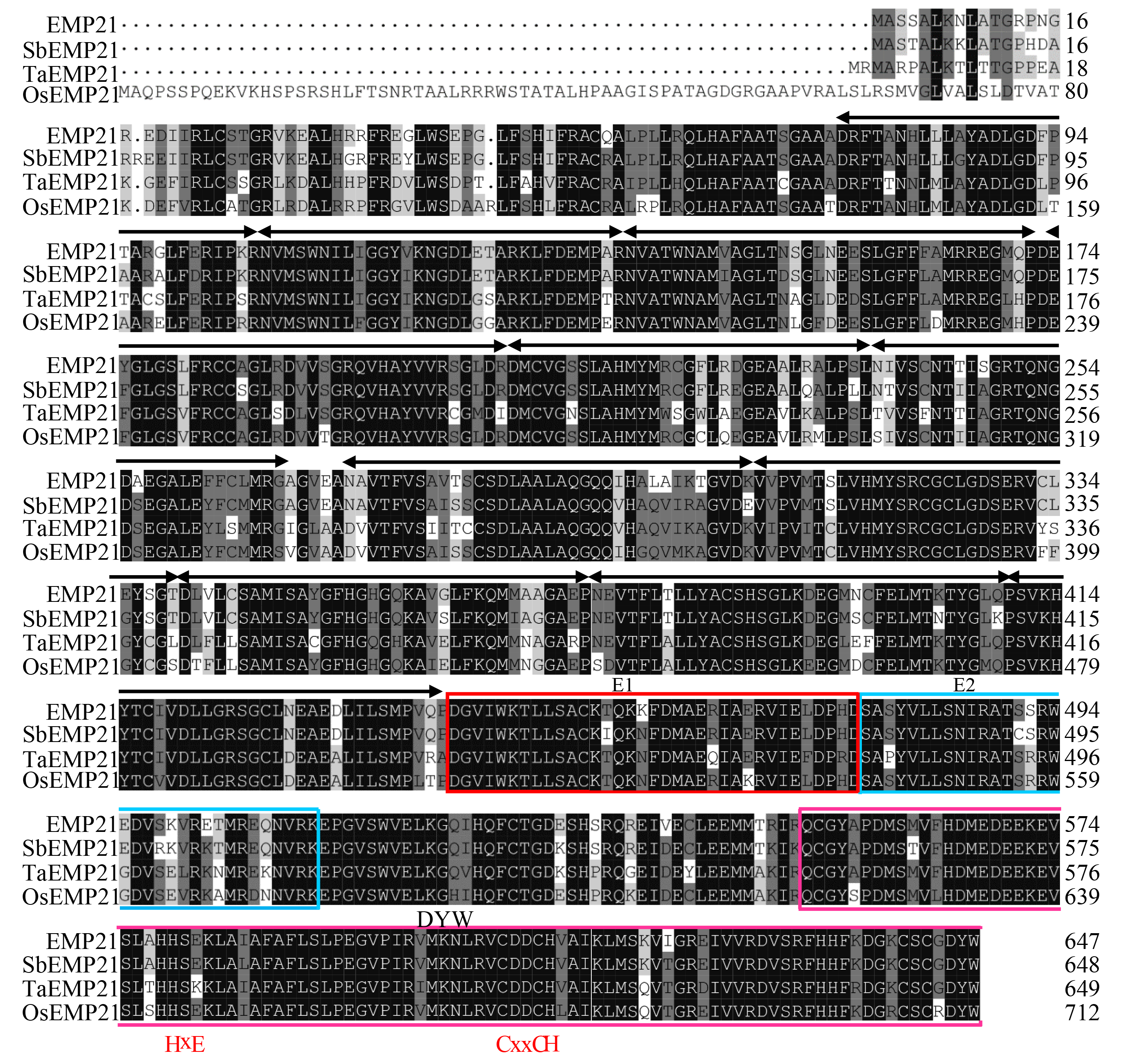

Supplement: S4 Fig — (TIF) [file pgen.1008305.s008.tif]

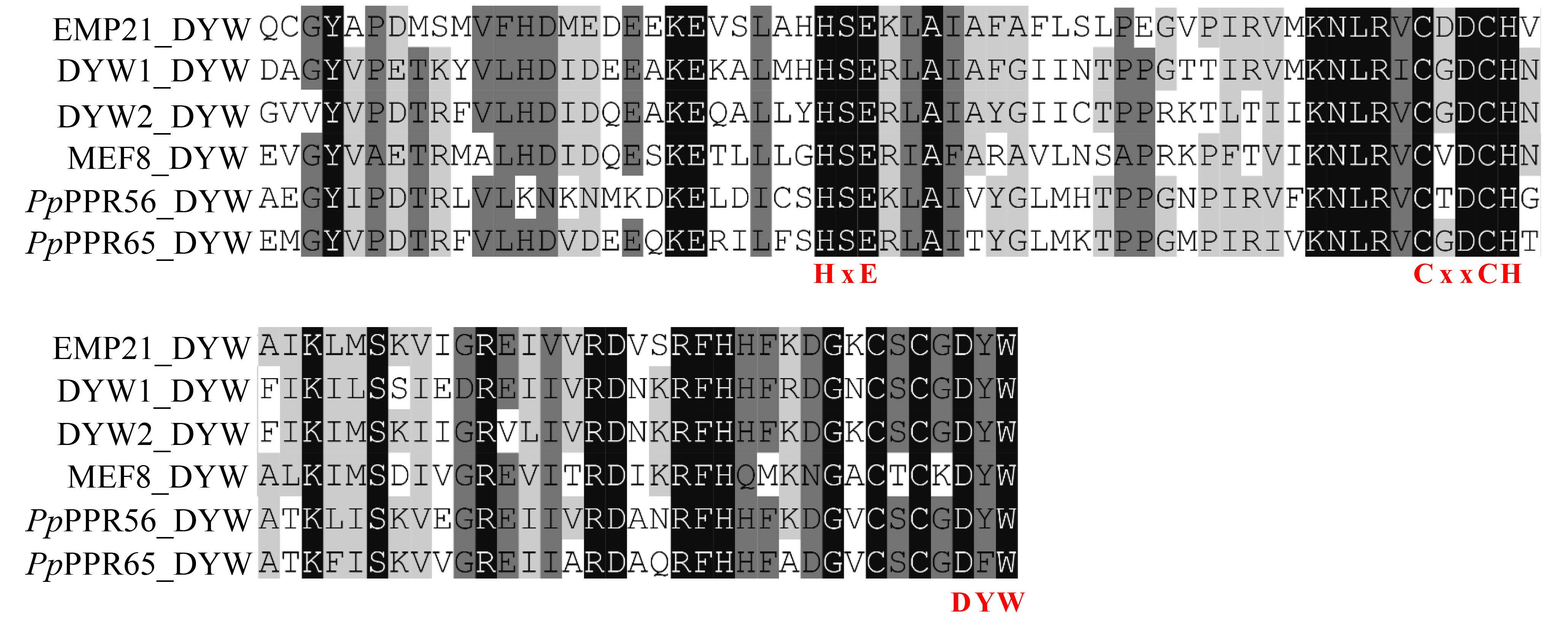

Supplement: S5 Fig — The conserved cytidine deaminase-like zinc binding signature residues HxE(x)nCxxC and the C terminal DYW tripeptide are shown in red font. (TIF) [file pgen.1008305.s009.tif]

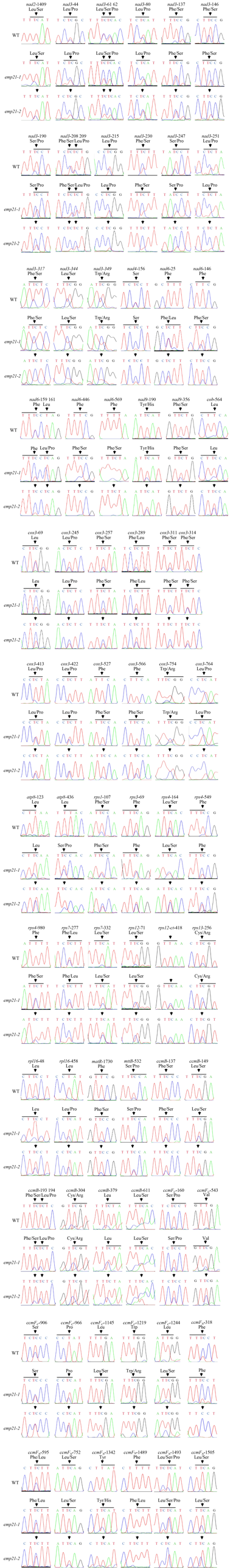

Supplement: S6 Fig — The defective editing sites are arrowed. The residues shown on the left are generated by an edited codon and those on the right by a non-edited codon. (TIF) [file pgen.1008305.s010.tif]

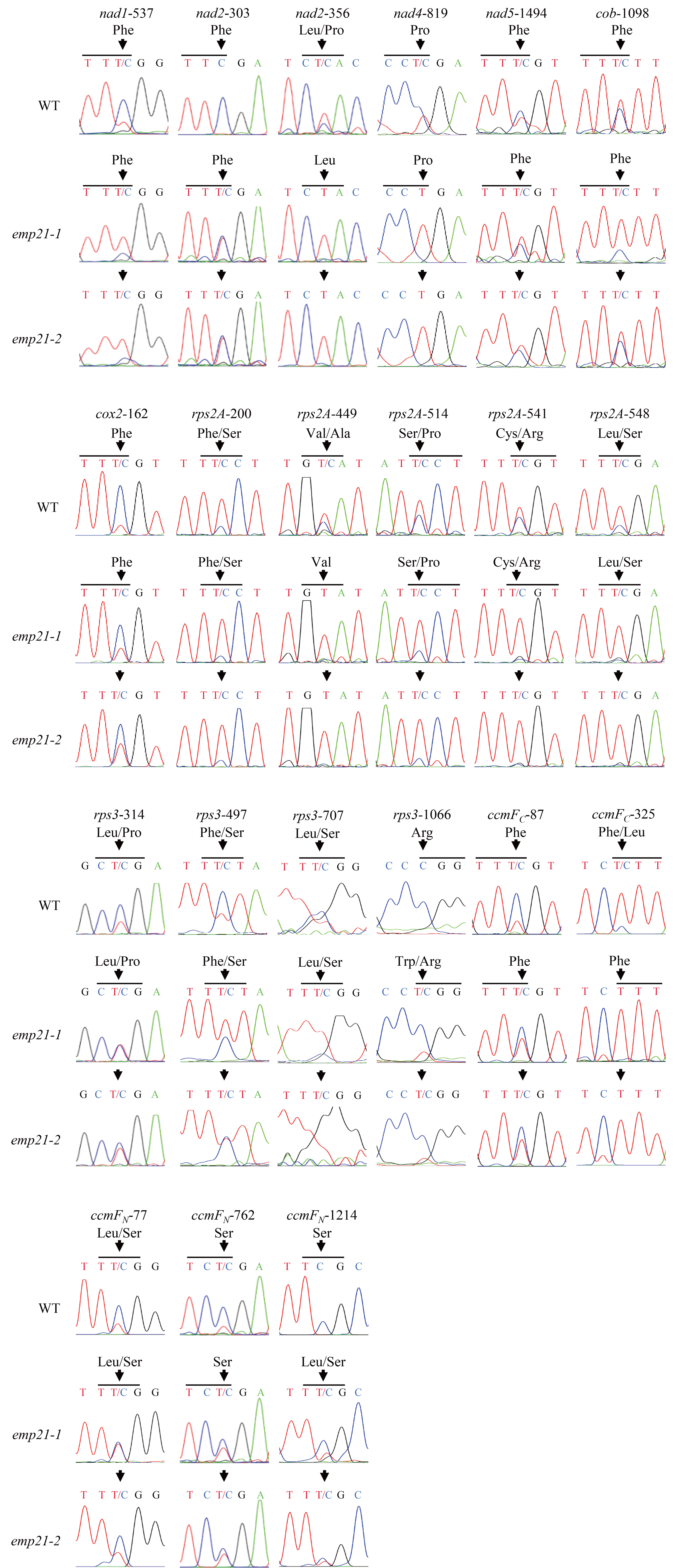

Supplement: S7 Fig — The increased editing sites are arrowed. The residues shown on the left are generated by an edited codon and those on the right by a non-edited codon. (TIF) [file pgen.1008305.s011.tif]

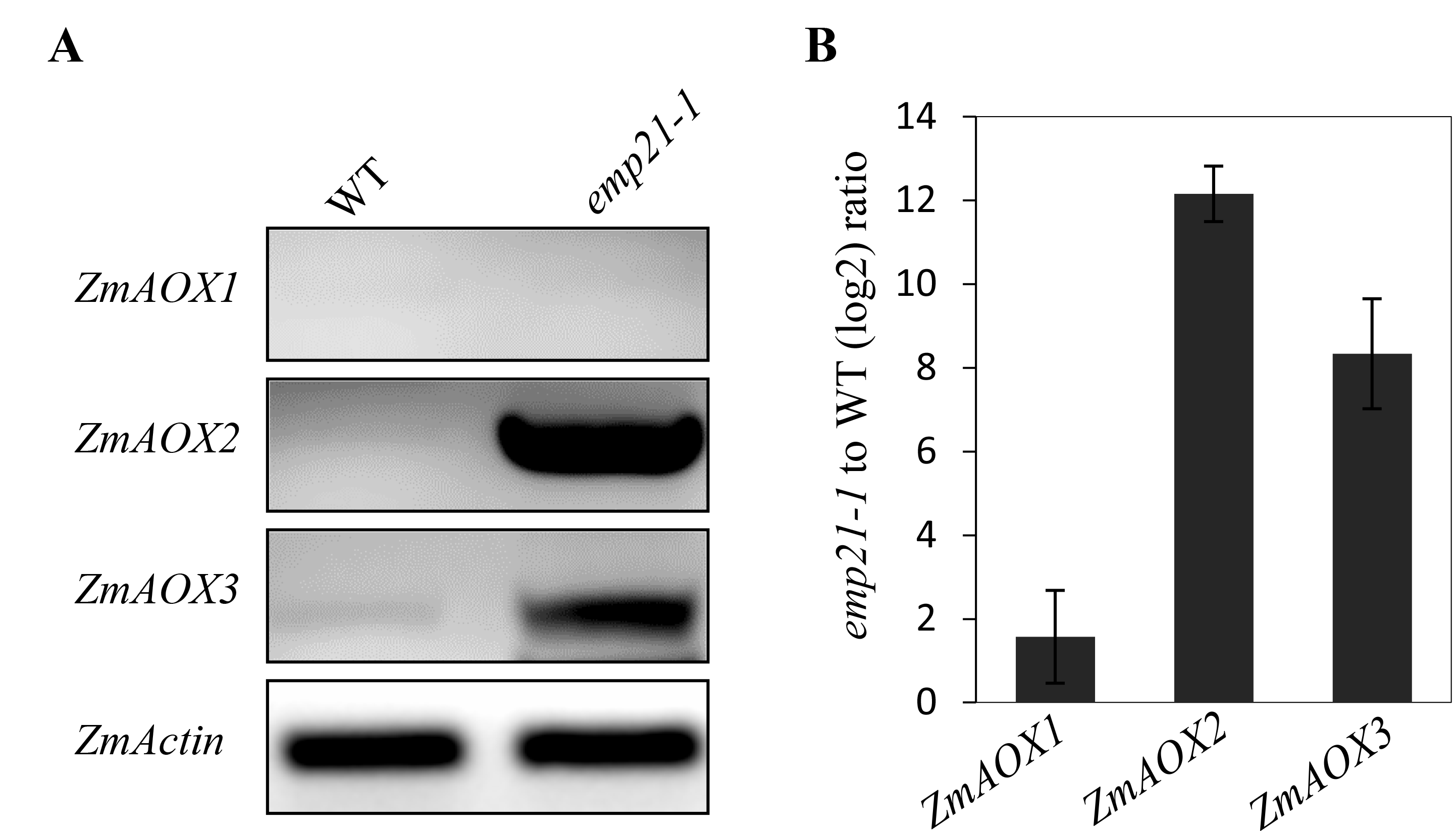

Supplement: S8 Fig — RT-PCR (A) and qRT-PCR (B) analyses of ZmAOX genes in WT and emp21-1. RNA was extracted from 12 DAP embryos and endosperms. qRT-PCR values represent three biological replicates and are normalized against ZmActin. Error bars represent the ±SD. (TIF) [file pgen.1008305.s012.tif]

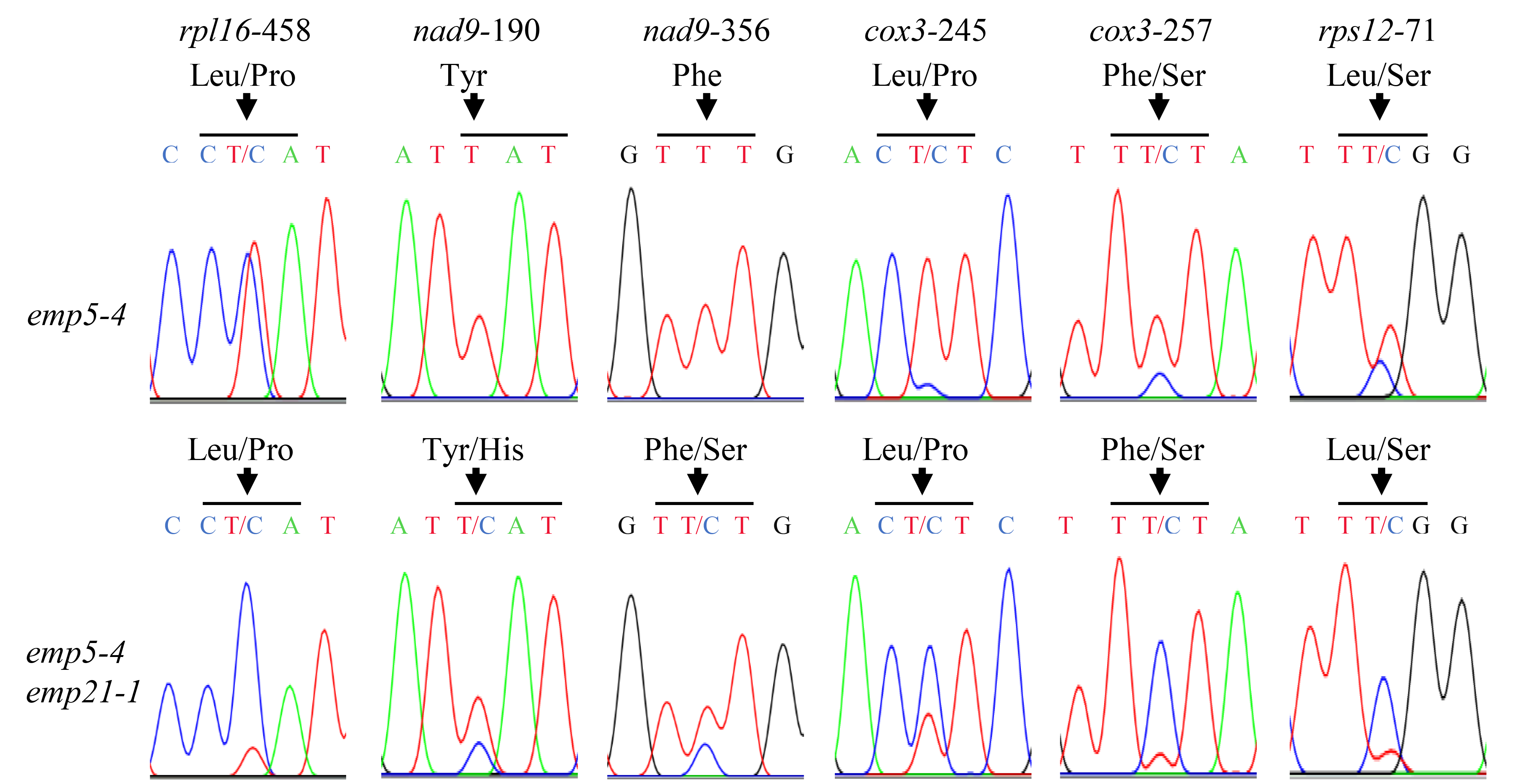

Supplement: S9 Fig — Defective sites are arrowed. The residues shown on the left are generated by an edited codon and those on the right by a non-edited codon. (TIF) [file pgen.1008305.s013.tif]

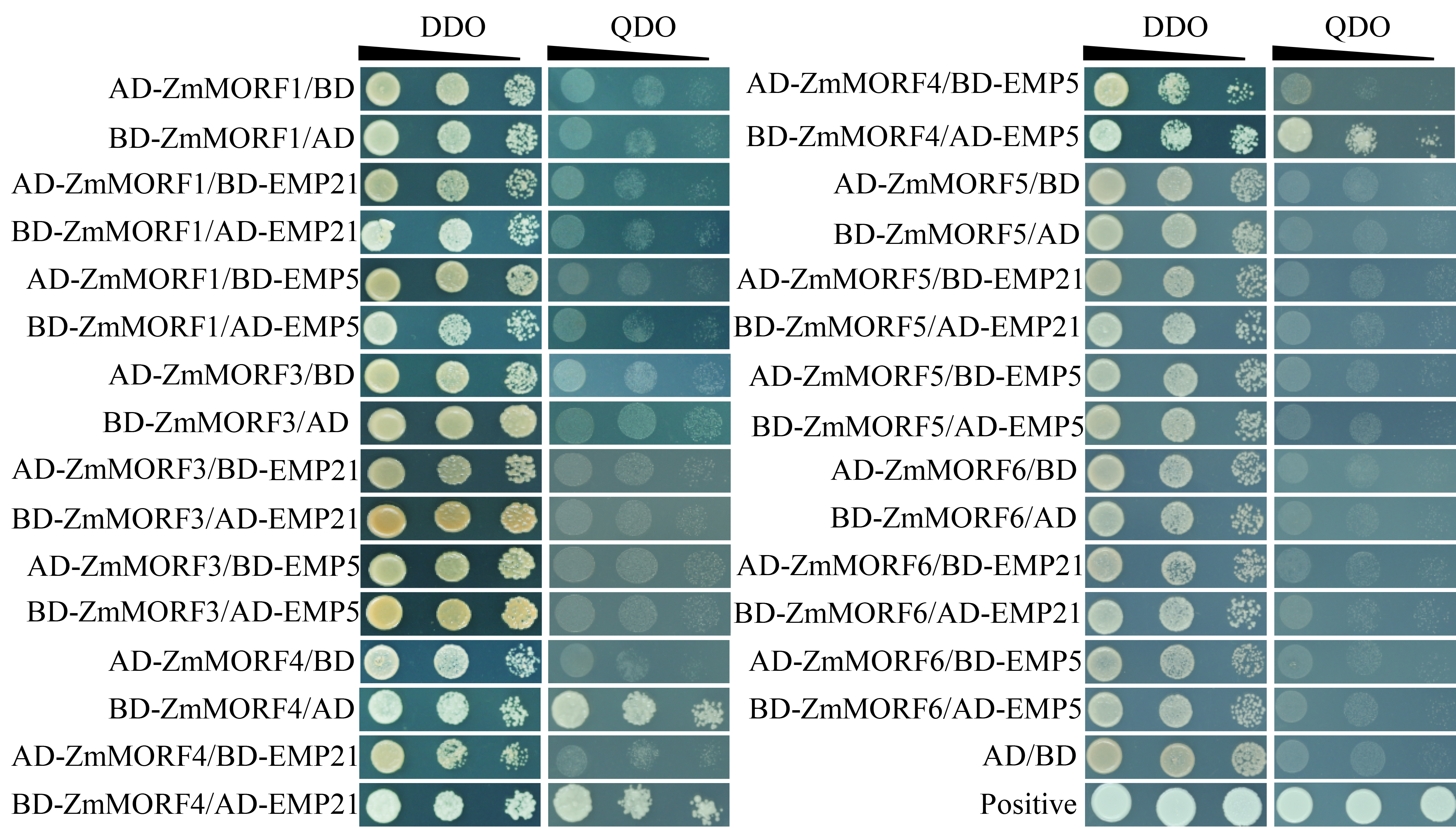

Supplement: S10 Fig — The colony pictures were taken after three days incubation at 30°C in SD/-Trp-Leu dropout (DDO) plates, as well as six days incubation at 30°C in SD/-Trp-Leu-His-Ade dropout (QDO) plates. (TIF) [file pgen.1008305.s014.tif]

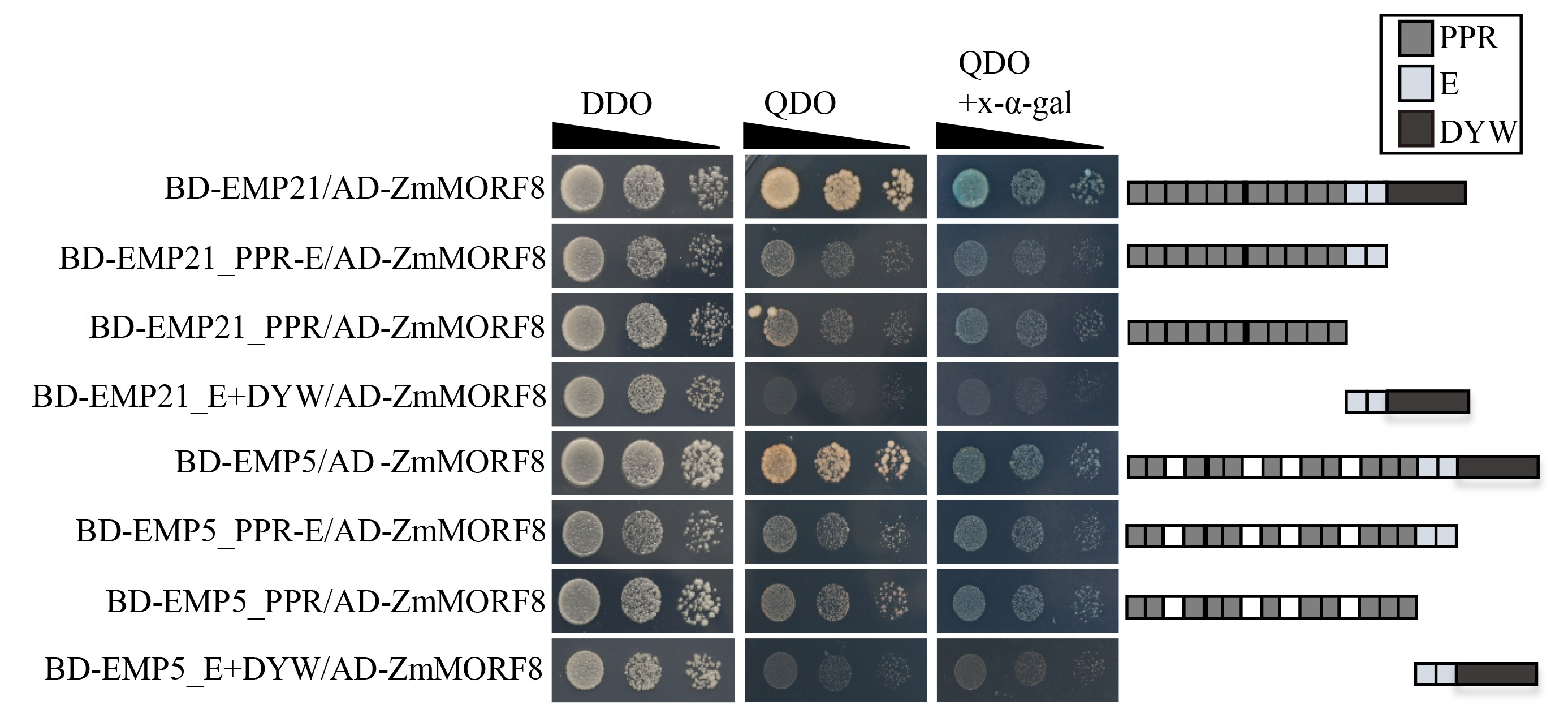

Supplement: S11 Fig — The colony pictures were taken after three days incubation at 30°C in SD/-Trp-Leu dropout (DDO) plates, as well as six days incubation at 30°C in SD/-Trp-Leu-His-Ade dropout (QDO) plates and SD/-Trp-Leu-His-Ade dropout + x-α-gal (QDO+ x-α-gal) plates. (TIF) [file pgen.1008305.s015.tif]

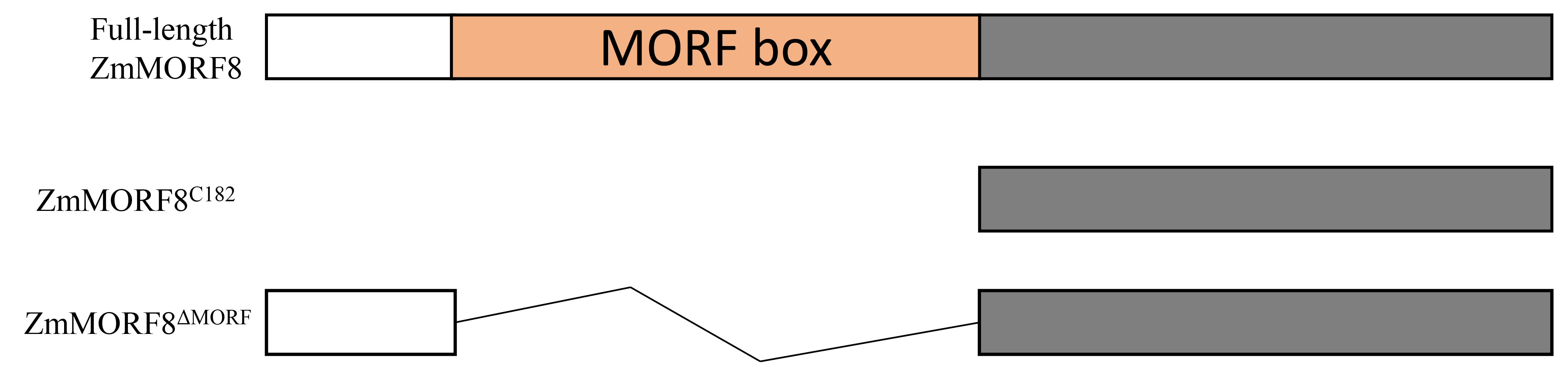

Supplement: S12 Fig — (TIF) [file pgen.1008305.s016.tif]
